# Towards Elucidating Structure–Spectra Relationships in Rhamnogalacturonan II: Computational Protocols for Accurate ^13^C and ^1^H Shifts for Apiose and Its Borate Esters

**DOI:** 10.3389/fmolb.2021.756219

**Published:** 2022-01-24

**Authors:** Vivek S. Bharadwaj, Luke P. Westawker, Michael F. Crowley

**Affiliations:** Renewable Resources and Enabling Sciences Center, National Renewable Energy Laboratory, Golden, CO, United States

**Keywords:** Quantum chemistry, Apiose, Borate, NMR chemical shifts, ^1^H and ^13^C, Gauge invariance approach, DFT, Rhamnogalacturonan II

## Abstract

Apiose is a naturally occurring, uncommon branched-chain pentose found in plant cell walls as part of the complex polysaccharide Rhamnogalacturonan II (RG-II). The structural elucidation of the three-dimensional structure of RG-II by nuclear magnetic resonance (NMR) spectroscopy is significantly complicated by the ability of apiose to cross-link *via* borate ester linkages to form RG-II dimers. Here, we developed a computational approach to gain insight into the structure–spectra relationships of apio–borate complexes in an effort to complement experimental assignments of NMR signals in RG-II. Our protocol involved structure optimizations using density functional theory (DFT) followed by isotropic magnetic shielding constant calculations using the gauge-invariant atomic orbital (GIAO) approach to predict chemical shifts. We evaluated the accuracy of 23 different functional–basis set (FBS) combinations with and without implicit solvation for predicting the experimental ^1^H and ^13^C shifts of a methyl apioside and its three borate derivatives. The computed NMR predictions were evaluated on the basis of the overall shift accuracy, relative shift ordering, and the ability to distinguish between dimers and monomers. We demonstrate that the consideration of implicit solvation during geometry optimizations in addition to the magnetic shielding constant calculations greatly increases the accuracy of NMR chemical shift predictions and can correctly reproduce the ordering of the ^13^C shifts and yield predictions that are, on average, within 1.50 ppm for ^13^C and 0.12 ppm for ^1^H shifts for apio–borate compounds.

## Introduction

Computational prediction of the spectroscopic chemical shifts of nuclear magnetic resonance (NMR) can significantly improve its capability as an essential technique for the identification and characterization of complex biomolecular structures in solution (Duus et al., 2000; Wüthrich, 2003; Bifulco et al., 2007; Lodewyk et al., 2012; Tantillo, 2013; Iron, 2017; Krivdin, 2019). NMR techniques play a vital role in the structural elucidation of one of the least structurally characterized classes of biological molecules—carbohydrates—since they cannot otherwise be characterized *via* methods such as X-ray crystallography (Van Halbeek, 1994; Duus et al., 2000; Toukach and Ananikov, 2013a). Carbohydrates have a diverse set of building block residues that can be tied together with a range of inter-residue linkages to form complex macromolecules of biological significance in plants and animals (Duus et al., 1999). Plants harness the sugar building blocks available in nature to build large, complex, and heterogenous polysaccharide structures that constitute the plant cell wall.

**FIGURE 1:**
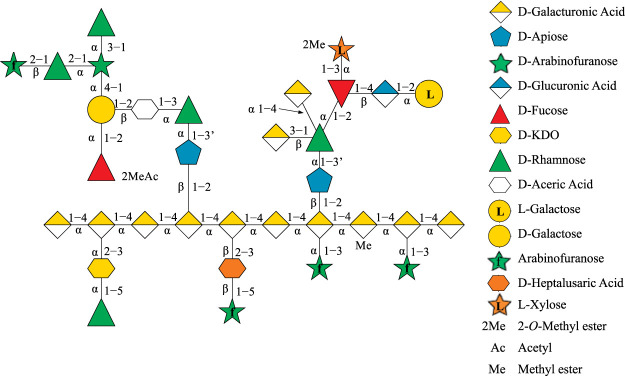
Topological structure of Rhamnogalacturonan II (RG-II) depicted as linked sugar molecules, with apiose as a *blue pentagon*.

Rhamnogalacturonan-II (RG-II) is a pectin molecule that exemplifies this phenomenon as the most complex polysaccharide known in nature, consisting of 12 monosaccharide units interconnected by 21 glycosidic linkages (Ndeh et al., 2017; O’Neill et al., 2020). Apiose is one of the 12 monosaccharide units in RG-II and has special significance as it is involved in dimerizing RG-II *via* a borate–ester cross-link (O'Neill et al., 1996; Bharadwaj et al., 2020). The formation of this borate–ester cross-link is required for normal plant growth, and most RG-II found in the plant cell wall remains cross-linked (O'Neill et al., 2004). The chemical structure and topology of RG-II (Figure 1) have only been recently determined by systematically deconstructing RG-II using a multitude of linkage-specific hydrolase enzymes (Ndeh et al., 2017). However, the three-dimensional (3D) structure of RG-II in solution in its monomeric and dimerized states still remains to be elucidated.

Characterizing the 3D structure of complex polysaccharides using NMR is challenging due to the myriad linkages between monosaccharides that often only differ from each other in stereochemistry (Duus et al., 2000). Furthermore, the diverse palette of available monosaccharides allows for structurally complex molecules, all of which share very similar chemical groups (hydroxyl groups and glycosidic linkages) resulting in NMR chemical shifts located in a much narrower spectral region than for proteins and nucleic acids (Toukach and Ananikov, 2013a), leading to overlaps in spectral data that limit the differentiation between specific residues within a polysaccharide (Bubb, 2003).

Computational approaches play an important role in overcoming these challenges. The use of empirical methods that are based on experimental chemical shift databases for spectral assignments may offer quick predictions, but are frequently inaccurate for carbohydrates (Pierens, 2014). The wide diversity of substructures and the complex 3D arrangements of polysaccharides far exceed the parameterization space employed for the development of these databases (Pierens, 2014). As a result, these methods are often unable to distinguish between diastereomers or account for interspatial coupling (Willoughby et al., 2014; Benassi, 2017), greatly hindering the elucidation of stereochemistry within a polysaccharide. On the other hand, quantum mechanical (QM) approaches to predicting NMR shifts have been more successful in aiding experimentalists to improve the reliability of NMR and correlate the spectra to the structure by predicting the chemical shift values of complex compounds (Pierens, 2014; Tarazona et al., 2018; Ahmed et al., 2020). This approach has even been used to correct previously misassigned spectra that relied on experimental databases and empirical-based predictions (Pierens, 2014).

QM-based approaches use the gauge-invariant atomic orbital (GIAO) method to calculate the isotropic magnetic shielding values from electronic structure calculations (Ditchfield, 1974; Schreckenbach and Ziegler, 1995). Early studies applied the GIAO approach to predict NMR shifts for small molecules and relied on Hartree–Fock (HF)- and post-Hartree–Fock (post-HF)-based methods (Schreckenbach and Ziegler, 1995; Helgaker et al., 1999). However, the high computational costs of the HF and post-HF-based *ab initio* methods for larger molecules, such as carbohydrates, have prompted investigations into using density functional theory (DFT) as a more tractable method (Taubert et al., 2005; Bagno et al., 2007; Toukach and Ananikov, 2013a). However, protocols that utilize DFT have been shown to have limitations (Teale et al., 2013; Benassi, 2017), with many previous studies having large discrepancies from experimental data, including being unable to correctly predict the ordering of chemical shifts (Taubert et al., 2005; Bagno et al., 2007), which is a vital piece of information for spectral analysis. Additionally, several studies have struggled to replicate either ^1^H or ^13^C data, often with proton data showing little variation despite changes in the levels of theory while carbon data were shown to vary greatly depending on the chosen level of theory (Benassi, 2017).

It has been shown that changing the specified density functional and basis set (FBS) combination can greatly affect the level of accuracy of a computational prediction (Pierens, 2014). Another important factor is accounting for the effect of solvent, which can be modeled using either implicit (Cramer and Truhlar, 1999) or explicit (Taubert et al., 2005; Bagno et al., 2007) solvation models in QM calculations, molecular dynamics (MD), and molecular mechanics (MM) simulations. Implicit solvation models approximate the presence of a solvent using a dielectric constant, thereby imparting a screening effect. While explicit solvation models may be considered to be intuitively more accurate, Bagno et al. demonstrated that the presence of explicit water molecules did not result in improved ^13^C and ^1^H shift predictions for glucose (Bagno et al., 2007). This may be attributed to the fact that, in explicit solvation models, the shielding constant calculations are affected by the conformational distribution of the hydroxyl and hydroxymethylene groups in glucose, which in turn are impacted by the presence of explicit solvent molecules, the number of solvation shells considered, and the inherent effects of the force fields considered for water and glucose. Hence, considering the additional computational costs and the lower accuracy of explicit solvation models for NMR shift calculations, implicit solvation models were considered in this study.

**FIGURE 2:**
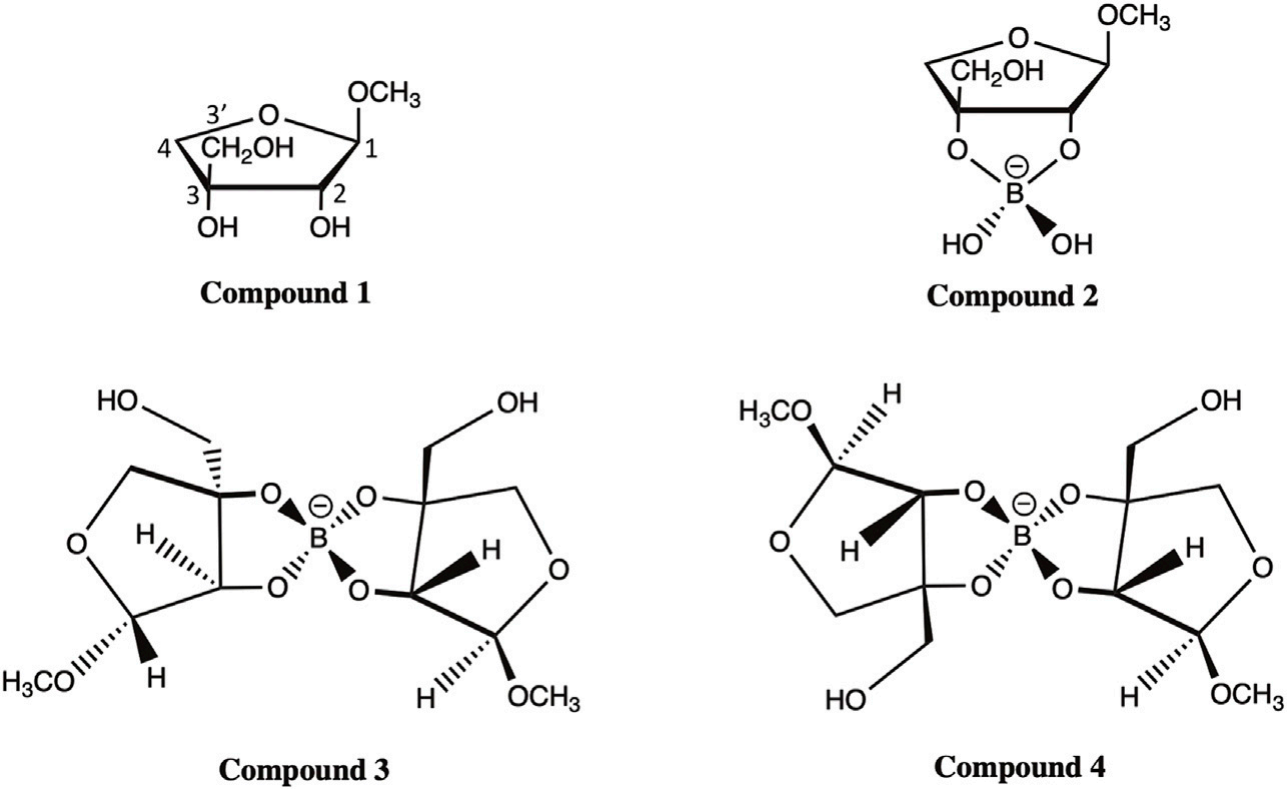
Compounds **1**–**4** of methyl apioside and apio–borate esters (Ishii and Ono, 1999).

Here, we applied the DFT approach and evaluated the accuracy of various FBS combinations and solvation models in predicting the chemical shifts of ^13^C NMR for four apiosyl compounds (Figure 2). The ability to accurately predict how the chemical shifts of apiose change upon dimerization with boric acid will aid experimentalists in using NMR to probe the dimerization process of RG-II. Therefore, this study is focused on apiose and its borate esters and is not a generalized study on the efficacy of these methods for all carbohydrates. Another reason for this focus is the fact that the experimental NMR shift data from Ishii and Ono (Ishii and Yanagisawa, 1998; Ishii and Ono, 1999) for apiose and its borate esters in D_2_O using methanol as the ^13^C NMR reference compound are the only data available for benchmarking the computational predictions presented here.

DFT calculations for chemical shift predictions involve two steps, a geometry optimization followed by an NMR shielding constant calculation, each of which may be performed using a specific FBS combination and a solvation model. Recent literature on NMR shift predictions in carbohydrates often has not accounted for solvation during geometry optimizations and has occasionally done so only during NMR shielding constant calculations (Bagno et al., 2007; Toukach and Ananikov, 2013b; Benassi, 2017). We evaluated 23 FBS combinations based on the levels of theory recommended in the literature for NMR chemical shift predictions and applied them using implicit solvation models and a reference correction to study apio–borate compounds. We demonstrate that considering implicit solvation during both the geometry optimization and the shielding constant calculation results in lower mean absolute errors (MAEs) for computational predictions with respect to the experimental data for apio–borate compounds. Additionally, we found that while considering solvation is important during both geometry optimizations and shielding constant calculations, there was a greater positive impact from using implicit solvation during geometry optimization for both charged and charge-neutral compounds. Across all 23 FBS combinations tested, we list the top-performing FBS combinations that had lower MAEs for ^13^C data than those reported in the literature for NMR predictions of carbohydrates (Taubert et al., 2005; Toukach and Ananikov, 2013b; Benassi, 2017), while also replicating the correct ordering of chemical shifts.

## Computational Methods

### Consideration of β-D-Apiosyl Compounds

All four apiosyl compounds studied here were capped with methyl groups on C1 and C3′ to resemble the connectivity of apiose to other carbohydrates within RG-II (Figure 1). The β-D-methyl apiofuranoside (compound **1**) and its borate diol monoester (compound **2**) and diesters (compounds **3** and **4**) are the same as those studied by Ishii and Ono (Ishii and Ono, 1999). During the dimerization process, compound **1** first formed a borate diol monoester (compound **2**), followed by the formation of stereo-isomeric borate diol diesters (compounds **3** and **4**), which were observed to be present in equimolar ratios (Ishii and Ono, 1999).

The observation of Ishii et al. that compound **1** makes up the majority of methyl apiofuranosides is consistent with the fact that β-D-apiose is the most prevalent form of apiose in nature (Ishii and Yanagisawa, 1998). While the β-D-apiose structure of compound **1** promotes dimerization, the ⍺-L-apiose structure of its diastereomer is a less common form of a methyl apiofuranoside that rarely undergoes dimerization partially due to steric hindrances during borate esterification, arising from the *trans*-orientation of the hydroxyl groups (Ishii and Ono, 1999). Although this is the case, we did predict the chemical shifts for methyl ⍺-L-apiofuranoside, which are discussed in *Section S1* of the Electronic Supplementary Material (ESM).

### Geometry Optimization and NMR Shielding Constant Calculations

The geometries of compounds **1**–**4** {methyl β-D-apiofuranoside; methyl 3-C-(hydroxymethyl)-β-D-threo-tetrofuranose 2,3-borate; bis[methyl 3-C-(hydroxymethyl)-β-D-threo-tetrofuranose]-(S)-2,3:2′,3′-borate; and bis[methyl 3-C-(hydroxymethyl)-β-D-threo-tetrofuranose]-(*R*)-2,3:2′,3′-borate} and the reference compounds (acetone and methanol) were optimized using Gaussian16 (Frisch et al., 2016) at the B3LYP/6-31+G(d,p) level of theory, unless otherwise noted. The convergence criteria and the thresholds for self-consistent field (SCF) calculations are listed in Supplementary Table S2. Complete convergence for optimized structures was ensured by a vibrational analysis that revealed no imaginary eigenfrequencies. Geometry optimizations were conducted under gas-phase conditions and in the presence of an implicit solvent using a self-consistent reaction field (SCRF) (Tomasi et al., 2005) with the optimized coordinates for all compounds listed in *ESM Section S10* and depicted in Supplementary Figure S5. This implicit solvation model used a polarizable continuum model with the integral equation formalism variant (IEFPCM) (Tomasi et al., 1999), for which a dielectric constant of 78.06 was specified to represent the D_2_O solvent used in the experimental conditions.

NMR shielding constant calculations were then conducted on each optimized geometry to yield an isotropic magnetic shielding value for each carbon and hydrogen atom. Unlike the geometry optimization for FBS, which was held constant, the various NMR FBS combinations listed in Table 1 were employed for the NMR shielding constant calculation, whose specific route lines are listed in Supplementary Table S3. These specified NMR FBS combinations were combined with two other independent variables: the solvation model for the optimization and the solvation model for the NMR shielding constant calculation.

**TABLE 1:** Nuclear magnetic resonance (NMR) functional–basis sets (FBS) 1–23 identified by their FBS combinations for NMR shielding constant calculations.

NMR FBS no.	Functional^a^	Basis set^a^	Literature reference for NMR predictions^b^	^13^C MAEs for Figures 4 and 5 (ppm)^c^
1	B3LYP (Becke, 1993)	6-31G(d)	Chełmecka et al. (2007), Pierens (2014), Benassi (2017)	1.50
2	B3LYP (Becke, 1993)	6-311+G(2d,p)	Pierens (2014), Willoughby et al. (2014)	2.28
3	B3LYP (Becke, 1993)	cc-pVDZ	Pierens (2014)	2.37
4	B3LYP (Becke, 1993)	aug-cc-pVDZ	Pierens (2014)	1.93
5	BMK (Boese and Martin, 2004)	6-31G(d)	Pierens (2014)	2.58
6	BMK (Boese and Martin, 2004)	6-311G(d)	Pierens (2014)	3.39
7	mPW1PW91 (Adamo and Barone, 1998)	6-311+G(2d,p)	Pierens (2014), Benassi (2017)	1.85
8	PBE0 (Adamo and Barone, 1999)	6-311+G(2d,p)	Pierens (2014), Benassi (2017)	1.87
9	WC04 (Wiitala et al., 2006)	6-31g(d)	Pierens (2014)	7.34
10	WP04 (Wiitala et al., 2006)	aug-cc-pvdz	Pierens (2014), Benassi (2017)	1.88
11	CAM-B3LYP (Yanai et al., 2004)	6-311+G(2d,p)	O'Neill et al. (1996)	2.13
12	mPW1LYP	6-311+G(2d,p)	O'Neill et al. (1996)	2.13
13	B3LYP (Becke, 1993)	6- 311G(d, p)	O'Neill et al. (1996)	2.36
14	CSGT-LC-TPSSTPSS (Tao et al., 2003)	cc-pVTZ	Iron (2017)	1.95
15	PBE (Perdew et al., 1996)	6-311G**	Kemp (2016)	1.91
16	mPW1PW91 (Adamo and Barone, 1998)	6-31G(d)	O’Neill et al. (2020)	1.90
17	B3LYP (Becke, 1993)	TZVP	Taubert et al. (2005)	2.52
18	BP86	TZVP	Taubert et al. (2005)	3.27
19	B3PW91 (Becke, 1993)	6-31+G(d)	Kupka et al. (1999), Toukach and Ananikov (2013a)	1.72
20	B3LYP (Becke, 1993)	6-311G++(2d,2p)	Toukach and Ananikov (2013a)	2.31
21	PBE (Perdew et al., 1996)	TZ2p	Belyakov and Ananikov (2011), Toukach and Ananikov (2013a)	2.04
22	WC04 (Wiitala et al., 2006)	aug-cc-pVDZ	—	5.91
23	WP04 (Wiitala et al., 2006)	6-31G(d)	—	1.79

MAEs, mean absolute errors.

a Specific route lines are listed in Supplementary Table S3.

b Use of FBS in the literature for predicting chemical shifts.

c MAEs for ^13^C predictions using implicit solvation during both geometry optimizations and shielding constant calculations.

### Chemical Shift Calculations

The computed isotropic magnetic shielding values from the shielding constant calculation resembled the unreferenced NMR data found in the experimental analysis. Consequently, reference compounds were needed to convert the computed isotropic magnetic shielding values into NMR chemical shift data (Taubert et al., 2005), so as to be comparable to the experimental data reported in parts per million (Ishii and Ono, 1999). The choice of a reference did not change the relative ordering of shifts as the reference approach applied a constant correction value to all isotropic magnetic shielding values. Our initial results were consistent with the findings of other studies that tetramethylsilane (TMS) alone does not serve as a consistently good reference for NMR predictions (*ESM Section S4*) (Sarotti and Pellegrinet, 2009). Therefore, all our computational shifts were referenced to the experimental reference compounds used by Ishii and Ono (1999) (i.e., methanol for ^13^C and acetone for ^1^H), which are also structurally representative of the compounds being studied (Pierens, 2014; Taubert et al., 2005; Sarotti and Pellegrinet, 2009; Kupka et al., 1999). The reference correction values are reported in Supplementary Table S4. For every permutation of the solvation model and the NMR FBS combination that was run on a methyl apioside and its borate ester derivatives, the identical NMR FBS was also run on the appropriate reference compound (Taubert et al., 2005).

Equation 1 (see *ESM Section S2* for derivation) (Pierens, 2014; Sarotti and Pellegrinet, 2009) was used to calculate the computed chemical shift (*δ*_comp_) value for our compound. This was done by first comparing the computed isotropic magnetic shielding value of the second reference (*σ*_ref_) to its known experimental value (*δ*_ref_) in order to yield a reference correction (Supplementary Table S4) (Fulmer et al., 2010), from which we then subtracted the isotropic magnetic shielding value compound of interest (*δ*_comp_) to obtain its chemical shift value (*σ*_comp_).

$$\delta_{comp}=\sigma_{ref}+\delta_{ref}-\sigma_{comp}$$ (1)

**FIGURE 3:**
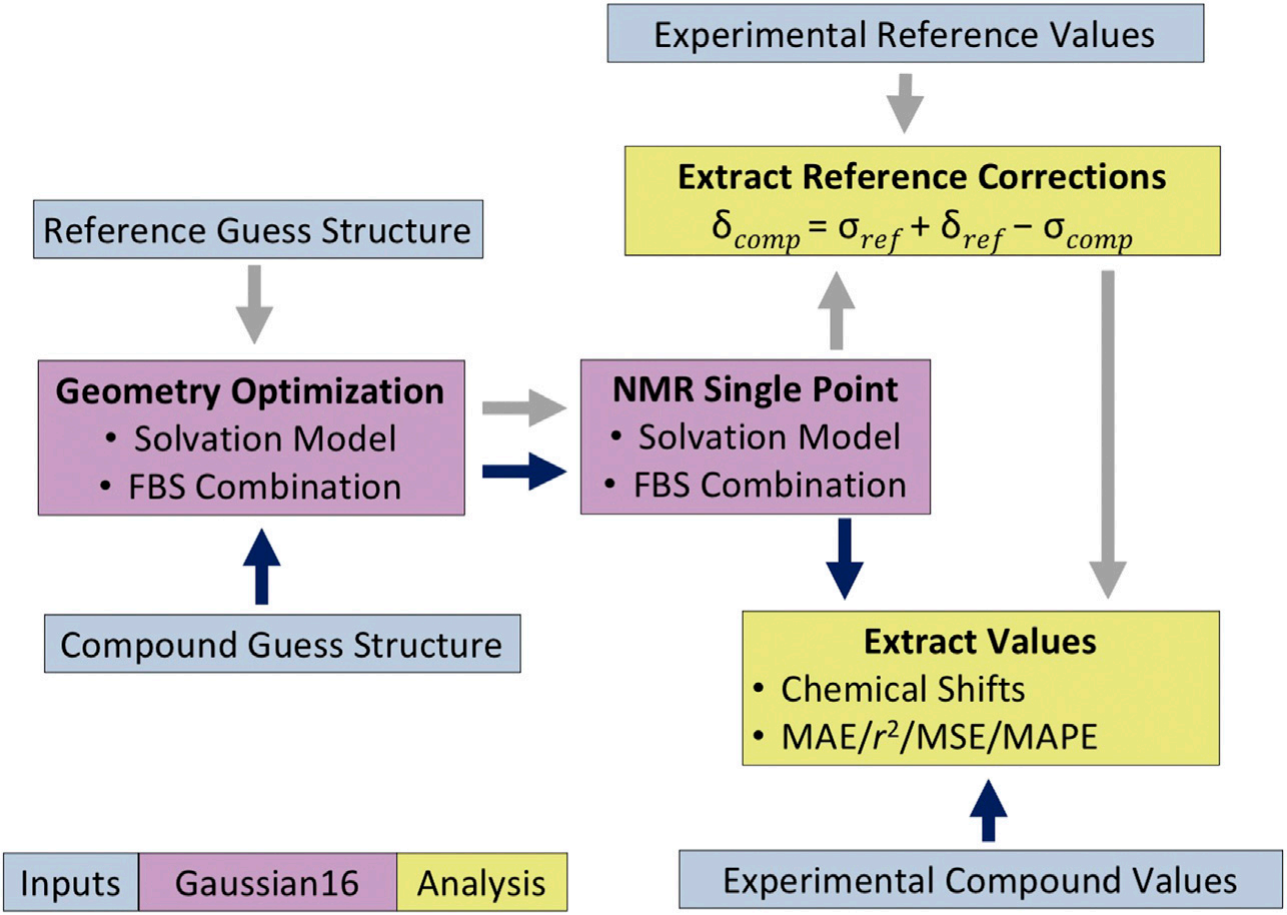
Protocol for quantum mechanical (QM)-based nuclear magnetic resonance (NMR) shift calculations. The *dark blue* and *gray arrows* indicate the analysis methodology for the compound of interest and the reference compound, respectively.

For compounds **3** and **4**, which are dimers, the computed isotropic magnetic shielding values reported were averaged over the two analogous atoms from each monomer before applying the reference correction. The ^1^H data for methyl peaks were averaged in a similar way. All statistical analyses were performed on these averaged chemical shifts. The overall protocol of how the chemical shift values were calculated is summarized in Figure 3.

### Statistical Analysis of FBS and Solvation Model Performance

To assess the efficacy of each FBS combination and solvation model, we used a variety of statistical analyses for determining the precision and accuracy of a chemical shift prediction. The primary tool was the MAE reported in parts per million, which measured the difference between the computed chemical shift value and the known experimental value (Ishii and Ono, 1999). The MAE for a given NMR FBS averaged the absolute errors of every atom on all four compounds. The mean absolute percent errors (MAPEs) were then used to compare the accuracy of the heteronuclear NMR predictions, as the MAE values were incomparable between ^1^H and ^13^C predictions due to differences in the magnitude of the chemical shifts. The MAE metric was contrasted with the mean signed error (MSE), which averaged all errors using signed values so as to measure whether a NMR FBS consistently over- or underpredicted the shift values, as observed by Taubert et al. (2005). Standard deviations (SDs), reported in parts per million, were calculated using such signed errors. Lastly, *r* ^2^ values were calculated by comparing the computed chemical shift values of each atom on compounds **1**–**4** with the experimental values. A low SD indicates high precision, also indicated by a high *r* ^2^ value. A smaller MSE indicates high accuracy. A low MAE or MAPE value tends to indicate both adequate precision and accuracy.

## Results

### Impact of Implicit Solvation Only During NMR Shielding Constant Calculations

**FIGURE 4:**
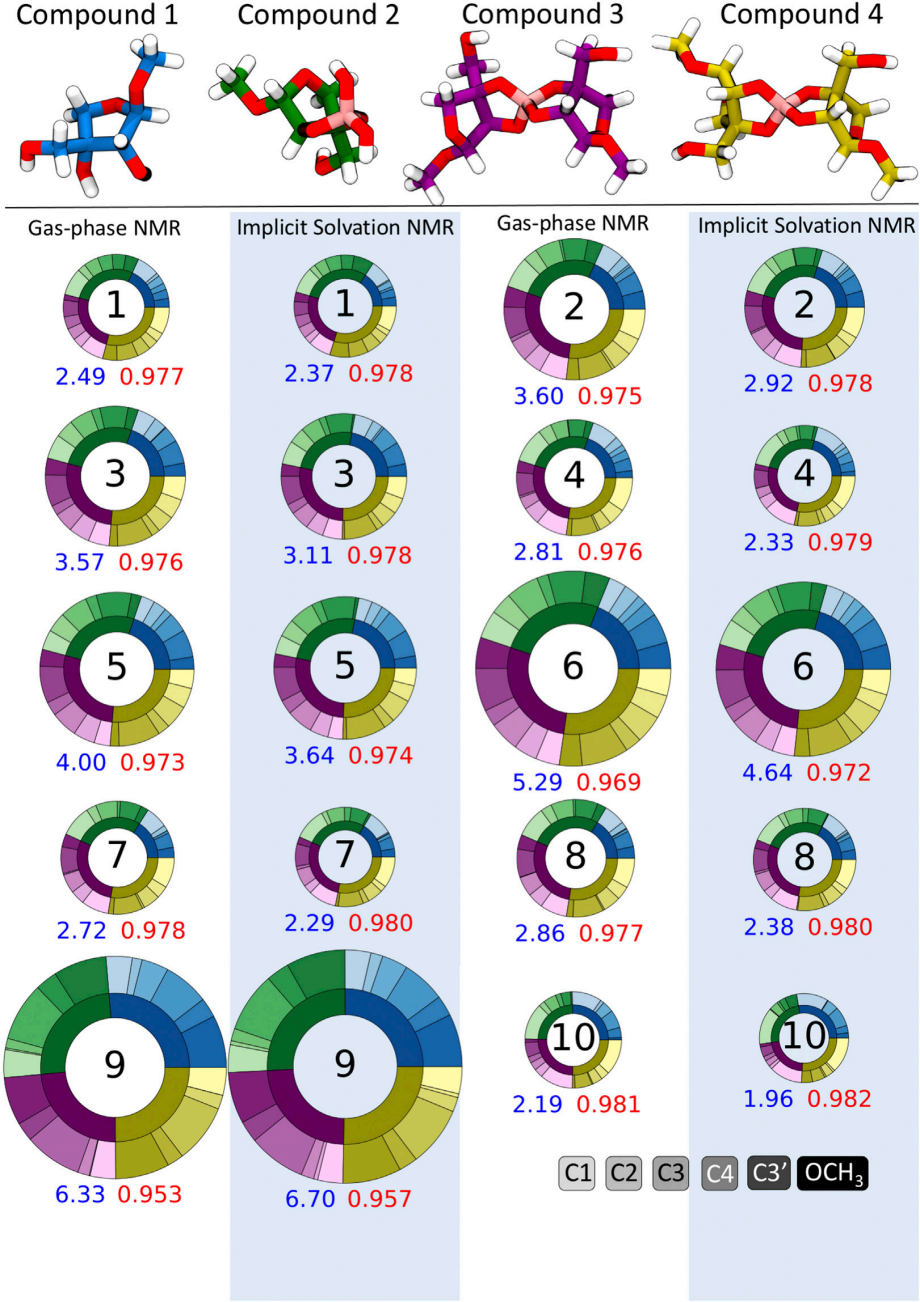
Mean absolute errors (MAEs) in parts per million for ^13^C nuclear magnetic resonance (NMR) predictions for NMR functional–basis sets (FBS) 1–10 represented as donut plots for gas-phase geometry optimizations. The donut size reflects the averaged MAE for each NMR FBS. The four colors within each donut indicate errors for each compound, and the shades within each color indicate errors on a specific carbon atom. MAEs and r^2^ values are shown in blue and red, respectively. Donuts on white and blue backgrounds indicate NMR values calculated in the gas phase and with implicit solvation, respectively.

Apiose and its charged borate esters were experimentally characterized in the presence of the solvent D_2_O (Ishii and Ono, 1999). To quantify the impact of considering solvation during the NMR shielding calculations, we predicted ^13^C shifts using NMR FBS 1–10, with and without implicit solvation during this NMR calculation. Figure 4 depicts circular plots (donut plots) that reflect the errors with respect to the experimental ^13^C NMR data for all six carbons in compounds **1**–**4** for these 10 NMR FBS combinations with their respective solvation model. For this comparison, all geometry optimizations were run in the gas phase without implicit solvation. Figure 4 shows that the MAEs ranged from 1.96 to 6.70 ppm, with the best performance by NMR FBS combinations 10, 4, 7, 8, and 1 with implicit solvation. The NMR computations that considered implicit solvation *via* IEFPCM (highlighted in blue) primarily resulted in MAEs lower than those of their counterparts run in purely gas-phase conditions without any solvation (white background).

Figure 4 shows that most NMR FBS combinations performed similarly across all four compounds, with errors relatively evenly split between compounds (each compound accounted for about a quarter of the total error). While the error was not evenly split between carbon atoms, there was no consistent trend about which atoms had the highest errors. In some cases, carbons C1 and OCH_3_ had higher errors, but they did not affect the shift ordering as they were the farthest upfield and farthest downfield chemical shift values, respectively.

### Impact of Implicit Solvation During Both Geometry Optimization and NMR Shielding Constant Calculations

While MAEs provide insights into the average accuracy of an FBS and solvation model, they do not show whether the shifts are in the correct order, which is an important aspect of NMR characterization. The experimental data in Table 2 indicated the following order toward the downfield direction for both methyl apiose and its borate derivatives: OCH_3_, C3′, C4, C2, C3, C1. The use of implicit solvation during the NMR shielding constant calculations improved the shift ordering and decreased the MAEs. However, all calculations that used gas-phase geometry optimizations, regardless of the FBS or solvation model used in the NMR shielding constant calculations, were unable to perfectly replicate the ordering of the ^13^C shift data. Table 2 shows that the orders of C2 and C3 for gas-phase geometry predictions were switched in the apio–borate compounds **2**–**4** compared with the experimental shift values. Incorrect ordering was observed for all other NMR FBS combinations for C2/C3 in at least one of the compounds.

**TABLE 2:** ^13^C shifts for compounds **1**–**4** from Ishii and Ono (1999) compared to the computationally predicted shifts using either gas phase or implicit solvation (IEFPCM).

Compound	Solvation model		Chemical shifts (ppm) (downfield → upfield)^b^						MAE^c^
	Optimization	NMR (FBS 1)^a^	C1	C3	C2	C4	C3′	OCH_3_	
**1**	–	Ishii et al.	110.20	80.08	77.30	74.32	64.23	56.78	–
	Gas phase	IEFPCM	108.53	79.62	78.76	73.17	64.47	52.73	1.50
	IEFPCM	IEFPCM	108.38	79.25	78.59	73.04	63.26	53.35	1.60
**2**	–	Ishii et al.	110.70	86.92	83.42	75.08	65.38	55.08	–
	Gas phase	IEFPCM	107.08	84.03	85.83	77.21	66.50	50.30	2.82
	IEFPCM	IEFPCM	110.41	83.92	83.30	72.33	64.20	52.24	1.70
**3**	–	Ishii et al.	110.18	87.09	83.25	74.86	65.13	55.06	–
	Gas phase	IEFPCM	108.45	84.92	85.85	76.95	66.43	50.96	2.33
	IEFPCM	IEFPCM	109.90	85.71	84.37	73.92	65.20	52.18	1.11
**4**	–	Ishii et al.	110.28	86.94	83.42	74.90	65.13	55.06	–
	Gas phase	IEFPCM	106.76	84.25	86.90	76.68	66.11	50.49	2.84
	IEFPCM	IEFPCM	110.48	85.39	84.24	72.16	63.64	52.22	1.61

IEFPCM, polarizable continuun model using the integral equation formalism variant; *FBS*, functional–basis set; *MAE*, mean absolute error.

a NMR shielding constant calculations using FBS 1 and implicit solvation (IEFPCM).

b Incorrect ordering of C3/C2 shifts as compared with the experimental data of Ishii et al. is highlighted in red.

c Accounting for implicit solvation during geometry optimizations decreases the MAEs.

**FIGURE 5:**
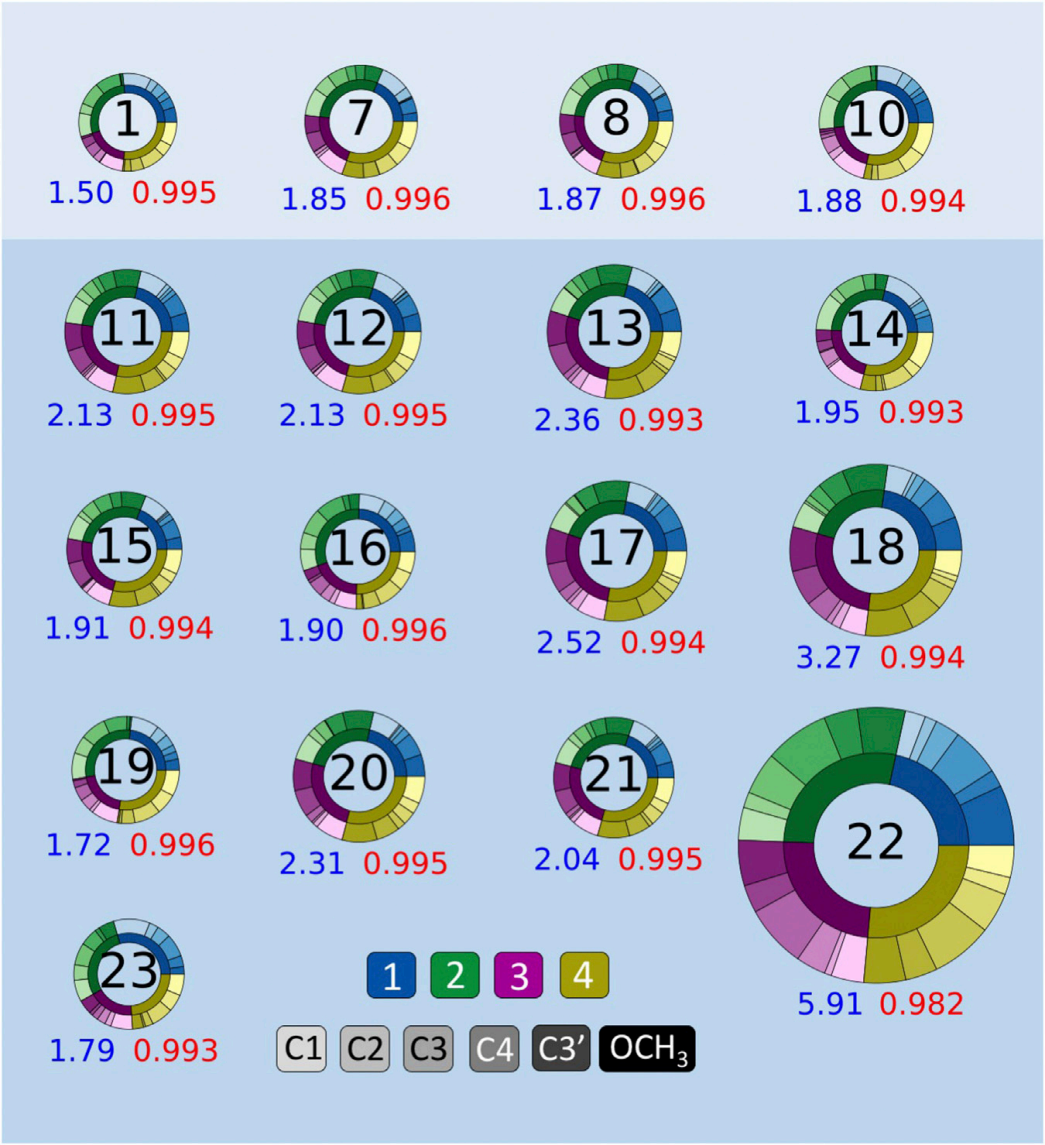
Mean absolute errors (MAEs, *blue*) in parts per million and *r* ^2^ values (*red*) for ^13^C nuclear magnetic resonance (NMR) predictions. NMR functional–basis sets (FBS) 11–23 are compared to the top-performing methods from NMR FBS 1–10, shown at the *top* (1, 7, 8, and 10). Donut plots for all NMR FBS 1-23 are shown in Supplementary Figure S2.

To ensure the correct ordering of the computationally predicted ^13^C shifts, we considered the impact of implicit solvation on geometry optimizations in addition to the NMR shielding constant calculations. The use of implicit solvation during both steps resulted in an ordering that was consistent with the experimental data for all compounds (Table 2). Additionally, we observed that using implicit solvation during both steps resulted in lower MAEs and increased *r* ^2^ values for all NMR FBS combinations than those for geometries obtained in the gas phase. Figure 5 depicts the improved MAEs and *r* ^2^ values for the four best-performing NMR FBS combinations out of the initial 10: 1, 7, 8, and 10.

### Determining the Best FBS Combinations for Accurate NMR Shift Predictions

Having established a protocol that consistently replicated the ordering of chemical shifts by using implicit solvation for both geometry optimizations and NMR shielding constant calculations, we then proceeded to evaluate other NMR FBS combinations by using our protocol to improve upon other FBS combinations used in the literature. We identified 13 other NMR FBS combinations that had been recommended in the literature for NMR predictions and that could be used with implicit solvation on all our compounds (Taubert et al., 2005; Toukach and Ananikov, 2013a; Willoughby et al., 2014; Kemp, 2016; Benassi, 2017; Iron, 2017). This additionally allowed us to probe how a change in functional or basis set affects the performance of an FBS for apio–borate compounds.

We tested the same functional and various basis set combinations; for instance, switching the basis sets of NMR FBS 9–10 yielded NMR FBS 22–23. NMR FBS 11–23 were compared in Figure 5 to our previous top 4 NMR FBS combinations: 1, 7, 8, and 10. Table 3 shows the statistical analyses of the new top 6 best-performing NMR FBS combinations (NMR FBS 1, 19, 23, 7, 8, and 16) based on the lowest MAE values when paired with the implicit solvation models for both geometry optimization and shielding constant calculation.

**TABLE 3:** Statistical analysis [mean absolute errors (MAEs), mean absolute percent errors (MAPEs), and *r* ^2^ values] of the performance of the top 6 nuclear magnetic resonance (NMR) functional–basis set (FBS) combinations for predicting ^13^C and ^1^H shifts using implicit solvation during both geometry optimizations and NMR shielding constant calculations.

NMR FBS no.	MAE (ppm)		MAPE (%)		*r* ^2^		MSE (ppm)		SD (ppm)	
	^13^C	^1^H	^13^C	^1^H	^13^C	^1^H	^13^C	^1^H	^13^C	^1^H
1	1.50	0.12	2.16	2.98	0.995	0.892	−1.21	−0.02	1.39	0.16
16	1.90	0.11	2.63	2.69	0.996	0.913	−1.80	−0.05	1.19	0.14
19	1.72	0.10	2.51	2.64	0.996	0.918	−1.52	0.00	1.43	0.14
23	1.79	0.13	2.53	3.44	0.993	0.927	−1.38	−0.11	1.56	0.13
7	1.85	0.13	2.61	3.44	0.996	0.893	−0.49	−0.06	2.19	0.16
8	1.87	0.14	2.60	3.48	0.996	0.888	−0.29	−0.06	2.26	0.16

The combination of low MAE values and low standard deviation (SD) between individual errors suggests both high accuracy and precision for NMR FBS 1 and 23.

MSE, mean signed error.

While we were able to improve our MAEs using different functionals for NMR shielding constant calculations, it was shown that our consistent use of the B3LYP functional for geometry optimizations (which was kept constant to limit the number of variables) yielded better results for NMR predictions than some suggested in the literature. Specifically, NMR FBS 15, 16, and 1 have been shown in the literature to work well with wB97XD, M062X, and wB97XD as geometry optimization functionals, respectively, when calculating chemical shifts for carbohydrates (Kemp, 2016; Benassi, 2017). The results in Supplementary Figure S3 show that these literature functionals for geometry optimizations performed worse than B3LYP.

The top 4 NMR FBS combinations for ^13^C predictions (Table 3) also performed the best at predicting ^1^H data (*ESM Section S7*). Due to the different magnitudes of the shift values of ^13^C and ^1^H, we used MAPEs to compare their ability to predict both ^13^C and ^1^H shifts. As seen in Table 3, the MAPE values for ^1^H were slightly larger than those for ^13^C, but NMR FBS 19, the best at replicating ^1^H data, had a MAPE of 2.65%, which was comparable to the corresponding 2.51% MAPE for ^13^C. The experimental chemical shift values for the protons within the apiosyl compounds were similar enough that our predictions can only replicate general trends, and we were unable to exactly reproduce the shift ordering for ^1^H. Even in the experiments by Ishii et al., they were unable to distinguish H3′a and H3′b and noted the miniscule differences, such as the 0.04-ppm difference between H2 and H4a and the 0.003-ppm difference for H1 in compounds **2**–**4** (Ishii and Ono, 1999).

### Exploring the Impact of Two Different Solvation Models

The aforementioned calculations were performed using the IEFPCM implicit solvation model (Tomasi et al., 1999). The SMD model (solvation model based on density) is an alternative implicit solvation model that considers more optimized radii for the bulk electrostatics contributions and has been demonstrated to result in more accurate solvation energies (Marenich et al., 2009). In order to explore whether the SMD solvation model provides further accuracy improvements to the NMR predictions for apiose and its esters, we calculated the ^13^C MAEs for the top 6 FBS combinations using an SMD solvation model. We observed that for all 6 FBS methods, SMD solvation resulted in improvements to the NMR predictions, with the most significant being for FBS 16, 1, 23, and 19 (MAE improvements of 0.36, 0.33, 0.33, and 0.29 ppm, respectively), while FBS 7 and 8 demonstrated only marginal improvements (MAE improvements of 0.06 and 0.03 ppm).

## Discussion

RG-II has numerous exotic sugar moieties that have not been extensively characterized through experiments or computational models, and the protocols recommended in the literature for NMR chemical shift predictions for carbohydrates remain to be evaluated for these sugars. Here, we have shown that common techniques, such as gas-phase geometry optimizations and the use of scaling factors to convert chemical shifts, lacked accurate predictive capabilities for the NMR chemical shifts of the apiosyl compounds of RG-II. Modeling of this system is complicated by the diastereomers, charged states, dimerized configurations, solvent interactions, and the effects of boron.

**FIGURE 6:**
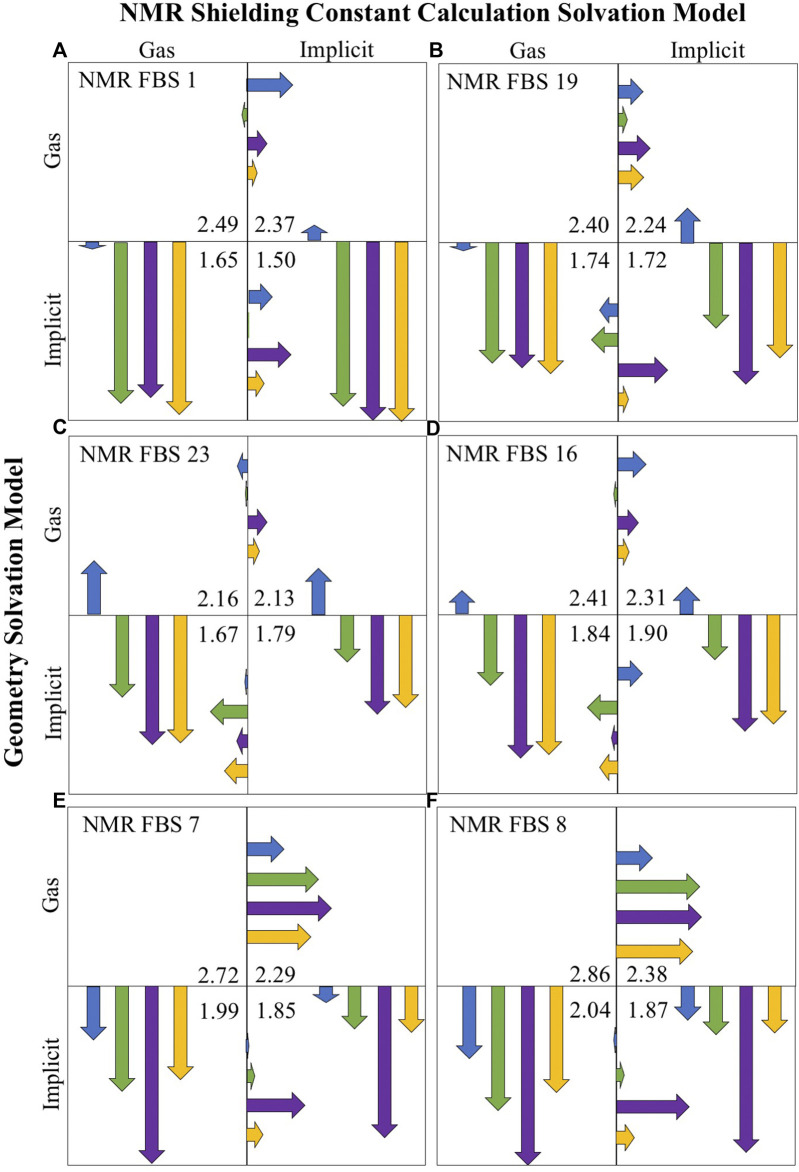
Mean absolute error (MAE) improvements upon solvation for the top-performing nuclear magnetic resonance (NMR) functional–basis sets (FBS): 1 **(A)**, 19 **(B)**, 23 **(C)**, 16 **(D)**, 7 **(E)**, and 8 **(F)**. Arrows are color-coded according to the compound number. Vertical arrows indicate MAE improvements due to the consideration of solvation during the geometry optimization, while horizontal arrows signify improvements during shielding constant calculations. The arrows point into the quadrant showing the most improvement. The listed MAE values (ppm) are the average of all compounds for that solvent combination. Their size indicates the size of MAE improvement upon a change in solvation model, where the full-length of a box is equal to an MAE improving by 1.23 ppm.

Figure 6 illustrates the impact of the consideration of solvation for geometry optimizations and NMR shielding constant calculations (with each quadrant representing a different solvation combination) for the four compounds of interest (indicated by the four different colors). For each NMR FBS combination, the vertical and horizontal arrows are quantitative representations of MAE improvements upon solvation for the geometry optimizations and shielding constant calculations, respectively. The arrows point to the protocol that enables the highest improvements in MAE for computational predictions. The vertical arrows, which are much larger, consistently point to the lower quadrants, showing the large and consistent benefit of using implicit solvation during geometry optimizations. Modeling implicit solvation benefits all compounds, particularly charged compounds **2**–**4**. The shorter horizontal arrows indicate a lower benefit of applying implicit solvation during shielding constant calculation.

It may be noted that based on the MAE value alone, NMR FBS 23 and 16 appeared to perform best in the lower left quadrant with gas-phase NMR shielding constant calculations. However, a closer look at the gas-phase NMR predictions (Supplementary Table S8) revealed an incorrect ordering of the shifts, with C2/C3 either being in the wrong order or being too similar to distinguish. When considering chemical shift ordering, the *r* ^2^ values, and SDs in addition to the MAE values across all four quadrants (Supplementary Table S9), the lower right quadrant (both implicit solvation) was the most accurate solvation combination for all NMR FBS.

While there are examples of NMR predictions of carbohydrates in the literature, these primarily studied uncharged compounds without solvent interactions. Our protocol, when compared with other studies in the literature, more accurately reproduced shift values for both charged and uncharged carbohydrates (Taubert et al., 2005; Ishii and Ono, 1999; Toukach and Ananikov, 2013b; Chełmecka et al., 2007; Boese and Martin, 2004). Taubert et al. utilized B3LYP/TZVP (NMR FBS 17) without solvation to predict the chemical shifts of a few saccharides (MAE = 2.28 ppm, *r* ^2^ = 0.97) (Taubert et al., 2005), but our top-performing NMR FBS coupled with implicit solvation for our compounds produced better MAE (1.50 ppm) and *r* ^2^ value (0.99) than those in their study. Additionally, Taubert’s top-performing method (NMR FBS 17 in this study) was not adept at modeling compounds **1**–**4**, as seen in Figure 5 (MAE = 2.89 ppm) (Taubert et al., 2005).

The accuracy of our top-performing NMR FBS 1 paired with implicit solvation can also be analyzed to report a root mean squared error (RMSD) of 1.82 ppm, which is equivalent to the MAE value of 1.51 ppm (Supplementary Table S6). This is an improvement over the use of B3LYP/6-31G(d) (NMR FBS 1), as done by Chelmecka et al., for studying glucopyranoside derivatives as their non-solvated gas-phase geometry optimizations and NMR shielding constant calculations yielded larger RMSEs (3–6 ppm) (Toukach and Ananikov, 2013b; Chełmecka et al., 2007). On the other hand, Kupka et al. reported a better RMSE value of 1.75 ppm during their study of glucose derivatives; they did not consider solvent effects when they used B3PW91/6-31+G* [comparable to B3PW91/6-31+G(d), NMR FBS 19] (Toukach and Ananikov, 2013b; Kupka et al., 1999). Applying NMR FBS 19 to our protocol showed that implicit solvation was required to correctly predict shift order (Supplementary Table S8). Belyakov et al. studied saccharides, but their use of PBE/TZ2p (NMR FBS 21) in gas-phase conditions without any solvation considerations yielded a higher RMSE of 2.43 ppm, and our study of NMR FBS 21 showed that it was not adequate at modeling compounds **1**–**4** (MAE = 2.04 ppm) (Belyakov and Ananikov, 2011; Toukach and Ananikov, 2013b).

Some of the protocols recommended in the literature used a different type of correction—the scaling factor correction—to convert computed isotropic magnetic shielding values to chemical shifts (Bagno et al., 2007; Pierens, 2014). This uses a computational method on a set of compounds, which are then compared to their experimental values so as to calculate scaling factors to convert computed isotropic magnetic shielding values to chemical shift data using a non-constant correction. Pierens et al. used scaling factors to predict experimental values with MAEs of <1.94 for ^13^C and 0.154 ppm for ^1^H for a non-carbohydrate compound using the same geometry optimization FBS of B3LYP/6-31+G(d,p) as in this study, but with non-solvated gas-phase optimizations, combined with NMR FBS 1–10, using a mix of gas-phase and implicit solvation models. We found that Pierens’s scaling factor values were unable to reliably predict the NMR data for compounds **1**–**4**, yielding errors of 2.5–8.8 ppm (Supplementary Table S10). Additionally, our protocol of modeling implicit solvation for geometry optimizations and shielding constant calculations followed by a constant reference correction method produced lower MAEs (30% lower for ^13^C and 15% lower for ^1^H) than those calculated using the scaling factor correction reported by Pierens.

We also quantified the effect of implicit solvation on the performance of the scaling factors, showing in Supplementary Table S10 that consideration of implicit solvation during both steps greatly reduced the MAEs and improved the relative shift ordering regardless of the correction method. Additionally, considering implicit solvation improved the reference correction method regardless of the reference compound. While using purely gas-phase conditions, TMS as a reference performed rather poorly compared to using methanol. However, as seen in Supplementary Table S5, the accuracy of using any reference greatly improved when implicit solvation was used during both steps, to the extent that TMS could yield lower MAEs than methanol for certain methods. As discussed in *Supplementary Information*, we continued to use methanol due to other benefits besides MAE and to match the experimental reference, as recommended in the literature (Kupka et al., 1999; Taubert et al., 2005; Sarotti and Pellegrinet, 2009; Pierens, 2014). This study demonstrated that implicit solvation improved predictions regardless of the correction method or the reference used.

Another type of correction uses an internal reference. Bagno et al. referenced the predicted chemical shifts of all carbon nuclei in glucose to the known isotropic magnetic shielding value of anomeric carbon instead of using a reference compound (Bagno et al., 2007). In addition to the limited applicability of internal references, the anomeric atoms in our study tended to have higher errors (Supplementary Table S6), making this approach unreasonable. Additionally, Bagno et al. only studied one compound, so their low MAE of 1.12 ppm was for one FBS that was optimized for one neutral sugar complex, just as our MAE for NMR FBS 1 for compound **3** was only 1.11 ppm. While Bagno et al. showed promise in using MD to take a sampling of 50–100 geometry conformations and averaging their chemical shift predictions, our NMR FBS 3–4 (similar to Bagno’s use of B3LYP/cc-pVTZ to reoptimize structures following MD simulations) did not perform well when applied to compounds **1**–**4**.

The metrics by which we measured the efficacy of a method can also guide improvements to the protocol. On the one hand, a well-performing protocol (i.e., low MAE value) that exhibited high precision (i.e., *r* ^2^ close to 1) but had low accuracy (i.e., a large MSE) was likely limited by constant systematic error that still was able to replicate the relative ordering of shifts. On the other hand, a well-performing protocol (i.e., low MAE value) that exhibited high accuracy (i.e., a small MSE) but had low precision (i.e., *r* ^2^ far from 1) was likely limited by a non-constant error that significantly hindered the applicability of the protocol for chemical shift predictions.

**FIGURE 7:**
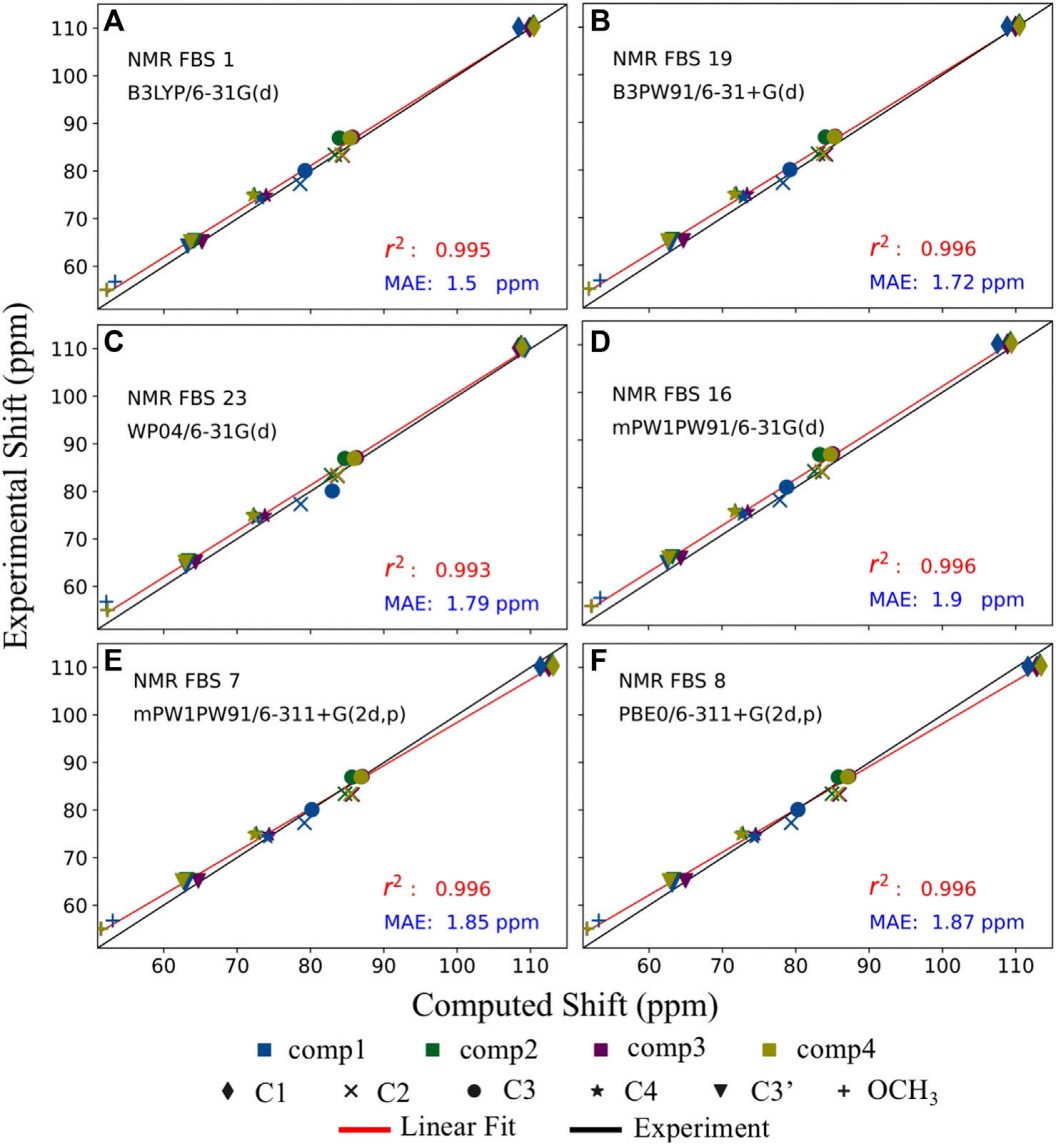
Balancing accuracy with precision. Comparison of the top 6 performing nuclear magnetic resonance (NMR) functional–basis sets (FBS): 1 **(A)**, 19 **(B)**, 23 **(C)**, 16 **(D)**, 7 **(E)**, and 8 **(F)**. Implicit solvation was modeled for both geometry optimizations and shielding constant calculations. Each of the six carbons is denoted by a different shape, while each of the four compounds is denoted by a different color. The black line represents the literature values for the experimental chemical shifts, whereas the red line represents the best-fit line for the computed chemical shifts used to extract the r^2^ values.

NMR FBS 16 exhibited high precision, as evidenced by the best *r* ^2^ value and the lowest SD, shown in Table 3 and Supplementary Table S9. However, out of all NMR FBS tested, 16 had the second largest MSE, which indicates very low accuracy. This can be observed in Figure 7 where the red linear best-fit line of NMR FBS 16 was significantly but consistently offset from the experiment’s black line. NMR FBS 16 is an example where the protocol predicting the relative shift values and ordering to high accuracy, but is unable to perfectly replicate the magnitude of the chemical shift.

Figure 7 illustrates that the computed values of the top 6 NMR FBS combinations were typically overpredicted compared to the expected experimental values, as seen by the red line (linear fit of the computed values) being above the black line. NMR FBS 8, on the other hand, had the best (smallest) MSE values while still maintaining one of the best *r* ^2^ values, suggesting high accuracy that came at the price of having a poor MAE value. Figure 7 shows that, unlike the constant offset displayed by NMR FBS 16, both NMR FBS 7 and 8 had different slopes from those of the experimental values, suggesting that they could benefit from a non-constant correction, such as the scaling correction method employed by Pierens (2014). This is consistent with Supplementary Table S10, where our application of Pierens’s non-constant scaling factors performed best for NMR FBS 7 and 8, with MAEs of just over 2.5 ppm. Conversely, the non-constant correction performed worst for NMR FBS 1, whose isotropic magnetic shielding values already showed a linear relationship with the experimental values and greatly benefited from a constant reference correction.

While some studies have suggested that a change in the basis set does not significantly change the predicted shift values (Benassi, 2017), our findings indicated that this does not apply for methyl apioside and its borate esters. While we did see an example of this behavior when the recommended NMR FBS of B3LYP/6-31G* (Chełmecka et al., 2007) produced indistinguishable chemical shifts from NMR FBS 1 that used B3LYP/6-31G(d), the majority of our results pointed to the importance of specifying a FBS combination as opposed to merely recommending a functional for chemical shift predictions. While NMR FBS 23 [WP04/6-31G(d)] was our third top-performing method with an MAE of 1.79 ppm, changing the basis set to aug-cc-pVDZ (NMR FBS 9) increased the MAE by 0.1 ppm, as seen in Figure 5. Conversely, changing the functional to B3LYP (NMR FBS 1) reduced the MAE by 0.29 ppm. Similar trends were observed when comparing NMR FBS 1–4, 13, 17, and 20, as they all shared the same functional (B3LYP) with different basis sets and thus yield various chemical shift predictions. Additionally, while the literature suggested that very large basis sets are needed to obtain accurate NMR predictions (Taubert et al., 2005), we did not find this to be the case for our system as our top-performing NMR FBS combinations did not have particularly large basis sets. These findings, in agreement with similar observations by Kupka et al. (2010) and Ahmed et al. (2020), highlighted the fact that generalized rules of using large basis sets do not inherently lead to accurate chemical shift predictions and thereby necessitate the evaluation of the efficacy of each combination.

## Conclusion

In this study, we evaluated the efficacy of various functional and basis set combinations and implicit solvation protocols for the accurate prediction of ^13^C chemical shifts for methyl apioside and its borate derivatives. In addition to the improved ^13^C shift predictions compared to those in recent literature on carbohydrates, we have been able to replicate the correct ordering of shifts for ^13^C data and distinguish between the apiofuranosides upon dimerization. We achieved this by considering implicit solvation during both the geometry optimization and the NMR shielding constant calculations. This protocol correctly predicted the same 6- to 7-ppm change in ^13^C chemical shift for C2/C3 that experimentalists used to distinguish between compound **1** and its borate esters (**2**–**4**) (Ishii and Ono, 1999).

Among the 23 FBS combinations that were assessed based on the SD values, *r* ^2^ analyses, and correct shift ordering, we observed four combinations (FBS 1, 16, 19, and 23) that all reproduced the correct ordering and were well within 2 ppm of the experimental values. We observed that our protocol was also able to demonstrate relatively accurate ^1^H predictions in most cases, with similar MAPE values to those observed for ^13^C. In some instances, the ordering of the ^1^H shifts was incorrectly predicted and could be attributed to the proton shifts of apiose being highly similar, a fact that was also experienced by experimentalists who struggled to distinguish them without using two-dimensional NMR (Ishii and Ono, 1999). Specifically, our protocol did not replicate the very precise 0.003-ppm change in the ^1^H chemical shift for H7 that experimentalists noted to distinguish between the borate esters (**2**–**4**).

From the RG-II context, in the future, in addition to being able to predict the ordering of ^1^H shifts to greater accuracy, we would also like to extend our protocol to predict chemical shifts in other sugar moieties such as galactose. We believe that this protocol can successfully be applied to study other complex RG-II mono- and oligosaccharides with NMR spectra that are difficult to assign and will play a key role in aiding our understanding of the role of specific sugars in the RG-II dimerization process.

In general, the use of NMR shifts to characterize non-covalent interactions and structural conformations is a crucial approach toward resolving the conformational ambiguities in complex organic molecules (Adams and Lerner, 1992; Siskos et al., 2015). The potential of computational predictions in elucidating structure–spectra relationships hinges on such detailed studies that are pivotal in enabling unique insights into the function and mechanisms of various chemical and biochemical systems.

## Data Availability

The original contributions presented in the study are included in the article/Supplementary Material, further inquiries can be directed to the corresponding author.
