# Supplementary material for: Towards Elucidating Structure–Spectra Relationships in Rhamnogalacturonan II: Computational Protocols for Accurate 13C and 1H Shifts for Apiose and Its Borate Esters
Source: Front Mol Biosci. 2022 Jan 24;8:756219. doi: 10.3389/fmolb.2021.756219 (PMC8820409; doi:10.3389/fmolb.2021.756219)
Supplement: Supplementary file 1 [file DataSheet1.docx]

Supplementary Information

for

Towards Elucidating Structure-Spectra Relationships in Rhamnogalacturonan II – Computational Protocols for Accurate ^13^C and ^1^H Shifts for Apiose and its Borate Esters.

Vivek S. Bharadwaj^a^*, Luke P. Westawker^a^, Michael F. Crowley^a^,

^a^Renewable Resources and Enabling Sciences Center, National Renewable Energy Laboratory, 15013 Denver West Parkway, Golden, Colorado 80401, USA

E-mail: *vivek.bharadwaj@nrel.gov

**Appendix A**

**Summary of SI data**

**Section S1: Consideration of methyl ⍺-L-apiofuranoside**

**Section S2: Calculating chemical shifts using a reference correction**

**Section S3: Specifications for Gaussian16 Calculations**

# **Section S4: Comparing the choice of reference compounds**

# **Section S5: Comparison of all NMR FBS combinations with implicit solvation during both steps**

**Section S6: Exploring the effect of other Geometry FBS combinations**

**Section S7: ^1^H NMR Shift predictions**

**Section S8: Effect of solvation model combination on shift predictions**

**Section S9: Calculating shifts using scaling factors**

**Section S10: Optimized geometry coordinates**

**Section S1****: Consideration of methyl ⍺-L-apiofuranoside**

While Ishii and Ono did not report experimental NMR values for compound 1’s diastereomer, methyl ⍺-L-apiofuranoside*,* in their 1999 paper where they presented chemical shifts for compounds 1- 4, they do report values in their 1998 paper.^1^


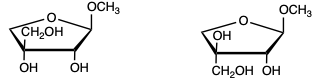


**Compound 1 Compound 5**

Figure S1: Structures for compound 1, methyl β-D-apiofuranoside (left), and its diastereomer, ⍺-L-apiofuranoside (right).

We investigated this compound as seen in Figure S1 in hopes of being able to computationally distinguish very similar structures such as diastereomers. While we were unable to perfectly match the 1998 experimental values for the structure that was labeled as compound 1’s diastereomer, their naming of the compounds appears to be inconsistent across the two papers. Table S1 shows that we could predict distinct values for the diastereomers in Figure S1, as we also did for the other diastereomers, compounds 3 and 4.

Table S1: Computed chemical shift values (ppm) for methyl ⍺-L-apiofuranoside (compound 5) compared to those of its diastereomer (compound 1). Computed using NMR FBS 1 with implicit solvation for both geometry optimizations and NMR calculations.

|  | Carbon # | | | | | |
| --- | --- | --- | --- | --- | --- | --- |
| Compound | 1 | 2 | 3 | 4 | 5 | 6 |
| 1 (E0 pucker state) | 107.98 | 79.92 | 81.35 | 74.23 | 66.60 | 52.50 |
| 5 | 106.40 | 79.06 | 82.27 | 74.45 | 65.80 | 52.52 |

While compound 1’s D-Apiose structure promotes dimerization, its diasteromer’s L-Apiose structure is a less common form of a methyl apiofuranoside that rarely undergoes dimerization partially due to its hydroxyl groups structurally hindering the bonding of the borate.^2^ As compound 1 and its borate esters are the most likely formations of apiose in RG-II, ^2^ we did not pursue further study into the chemical shifts of ⍺-L-apiofuranoside.

# **Section S2: Calculating chemical shifts using a reference correction**

While Equation 1 performs both a primary (TMS) and secondary correction, it has been simplified to only need the values of the second reference compound: methanol for ^13^C and acetone for ^1^H. The conversion equation takes the difference between the computed isotropic value and the known experimental chemical shift of the reference compound,^3^ which is then applied to the computed isotropic value of the compound of interest.^4^ Equation 1 was created by first using tetramethylsilane (TMS) as the primary reference, taking the difference between the computed isotropic value of TMS (σ_TMS_) and the literature value for its chemical shift (δ_TMS_) of 0 ppm,^3^ and then applying it to our compound’s isotropic value (σ_comp_). However, the TMS correction alone does not take into account that the experimental data was referenced to methanol or acetone, motivating a secondary correction using the appropriate reference that is structurally reminiscent of the compounds that is being predicted.^5^ For the secondary correction, we computed the isotropic value of the second reference (σ_ref_), methanol for ^13^C and acetone for ^1^H, then applied the TMS correction to compare it to the known literature value of the second reference (δ_ref_).^3^ In Equation 1, the isotropic values outputted by Gaussian are represented by sigma (σ) while all the shift values, including those in the literature as well as the shift value of the compound that is being calculated, are represented by delta (δ).

$$\delta_{comp}=\left( \left( \sigma_{TMS}- \delta_{TMS} \right)-\sigma_{comp} \right)-\left( \left( \sigma_{TMS}-\delta_{TMS} \right)-\sigma_{ref}-\delta_{ref}) \right)$$

$\delta_{comp}=\sigma_{ref}+\delta_{ref}-\sigma_{comp}$ (1)

The original TMS correction does not perfectly predict the chemical shifts of the second reference, allowing us to correct our computed data using a second reference that is electronically and structurally similar to our compound of interest as well as the same reference used in experiment. Equation 1 shows that combining the primary TMS correction with the secondary reference correction results in a simplified equation where mathematically all TMS variables cancel out, requiring us to model only the second reference.

For compounds 4 and 5, the shift values reported for the dimer are the averaged values for every two analogous atoms. Additionally, ^1^H data for methyl peaks are averaged across the chemical shifts of the three stationary hydrogens. All statistical analyses were performed on the averaged chemical shifts of such analogous atoms.

**Section S3: Specifications for Gaussian16 Calculations**

Table S2: Convergence criteria and thresholds for self-consistent field (SCF) calculations.

| Criteria | Threshold (hartree) |
| --- | --- |
| Maximum Force | 0.000015 |
| Maximum Displacement | 0.000060 |
| RMS Force | 0.000010 |
| RMS Displacement | 0.000040 |

Table S3: NMR FBS 1-23 identified by their functional-basis set combination for NMR SP calculations, along with their specified route lines. When the FBS were run with implicit solvation, “scrf(Read)” was added to their route line and “Eps=78.06” was added to the end of the file to specify the dielectric constant of D_2_O to model the implicit solvation.

| NMR  FBS # | Functional | Basis Set | NMR Route Line |
| --- | --- | --- | --- |
| 1 | B3LYP^30^ | 6-31G(d) | # nmr=giao B3LYP/6-31G(d) geom=allcheck guess=read scf=tight |
| 2 | B3LYP^30^ | 6-311+G(2d,p) | # nmr=giao B3LYP/6-311+G(2d,p) geom=allcheck guess=read scf=tight |
| 3 | B3LYP^30^ | cc-pVDZ | # nmr=giao B3LYP/cc-pVDZ geom=allcheck guess=read scf=tight |
| 4 | B3LYP^30^ | aug-cc-pVDZ | # nmr=giao B3LYP/aug-cc-pVDZ geom=allcheck guess=read scf=tight |
| 5 | BMK^32^ | 6-31G(d) | # nmr=giao BMK/6-31G(d) geom=allcheck guess=read scf=tight |
| 6 | BMK^32^ | 6-311G(d) | # nmr=giao BMK/6-311G(d) geom=allcheck guess=read scf=tight |
| 7 | mPW1PW91^33^ | 6-311+G(2d,p) | # nmr=giao mPW1PW91/6-311+G(2d,p) geom=allcheck guess=read scf=tight |
| 8 | PBE0^34^ | 6-311+G(2d,p) | # nmr=giao pbe1pbe/6-311+G(2d,p) geom=allcheck guess=read scf=tight |
| 9 | WC04 | 6-31g(d) | # nmr=giao blyp/6-31g(d) iop(3/76=1000007400,3/77=0999900001,3/78=0000109999) |
| 10 | WP04 | aug-cc-pvdz | # nmr=giao blyp/aug-cc-pvdz iop(3/76=1000001189,3/77=0961409999,3/78=0000109999) |
| 11 | CAM-B3LYP^36^ | 6-311+G(2d,p) | # nmr=giao CAM-B3LYP/6-311+G(2d,p) geom=allcheck guess=read scf=tight |
| 12 | mPW1LYP | 6-311+G(2d,p) | # nmr=giao mPW1LYP/6-311+G(2d,p) geom=allcheck guess=read scf=tight |
| 13 | B3LYP^30^ | 6- 311G(d, p) | # nmr=giao B3LYP/6-311G(d,p) geom=allcheck guess=read scf=tight |
| 14 | CSGT-LC-TPSSTPSS^37^ | cc-pVTZ | # nmr=CSGT TPSSh/cc-pVTZ geom=allcheck guess=read scf=tight |
| 15 | PBE^38^ | 6-311G** | # nmr=giao PBE1PBE/6-311G** geom=allcheck guess=read scf=tight |
| 16 | mPW1PW91^33^ | 6-31G(d) | # nmr=giao mPW1PW91/6-31G(d) geom=allcheck guess=read scf=tight |
| 17 | B3LYP^30^ | TZVP | # nmr=giao B3LYP/TZVP geom=allcheck guess=read scf=tight |
| 18 | BP86 | TZVP | # nmr=giao BP86/TZVP geom=allcheck guess=read scf=tight |
| 19 | B3PW91^30^ | 6-31+G(d) | # nmr=giao B3PW91/6-31+G(d) geom=allcheck guess=read scf=tight |
| 20 | B3LYP^30^ | 6-311G++(2d,2p) | # nmr=giao B3LYP/6-311++G(2d,2p) geom=allcheck guess=read scf=tight |
| 21 | PBE^38^ | TZ2p | # nmr=giao PBE1PBE/TZVP geom=allcheck guess=read scf=tight |
| 22 | WC04^35^ | aug-cc-pVDZ | # nmr=giao BLYP/aug-cc-pVDZ geom=allcheck guess=read scf=tight iop(3/76=1000007400,3/77=0999900001,3/78=0000109999) |
| 23 | WP04^35^ | 6-31G(d) | # nmr=giao BLYP/6-31G(d) geom=allcheck guess=read scf=tight iop(3/76=1000001189,3/77=0961409999,3/78=0000109999) |

# **Section S4: Comparing the choice of reference compounds**

The reference correction values and statistical analyzes presented below compare the three reference compounds when using implicit solvation for both geometry optimizations and NMR calculations, which was shown to yield the most accurate shift ordering. The constant reference corrections do not change the chemical shift ordering, how much a shift values changes between compounds, or how distinct the shifts of similar carbons are (i.e. C2/C3). As such, the choice of a reference compound primarily affects how similar in value the predicted shift values are to experimental value. The isotropic magnetic shielding constants of the reference compounds were computed under the same conditions as used to model the compounds and were then input into Equation 1 to extract the reference correct values listed below, which averaged the values of analogous atoms as done when extracting experimental reference values.

Table S4: Reference correction values for methanol, acetone, and TMS when using implicit solvation during both geometry optimizations and NMR calculations.

| Reference Correction Values (ppm) | | | | | |  |
| --- | --- | --- | --- | --- | --- | --- |
| Methanol | | Acetone | | TMS | |  |
| NMR FBS # | ^13^C | ^1^H | ^13^C | ^1^H | ^13^C | ^1^H |
| 1 | 188.65 | 32.024 | 192.17 | 32.336 | 190.20 | 32.183 |
| 2 | 177.02 | 31.523 | 181.50 | 31.805 | 183.11 | 31.826 |
| 3 | 191.92 | 31.369 | 194.39 | 31.701 | 194.09 | 31.519 |
| 4 | 188.81 | 31.271 | 192.15 | 31.561 | 192.52 | 31.686 |
| 5 | 194.09 | 31.994 | 194.81 | 32.153 | 196.76 | 32.069 |
| 6 | 182.93 | 31.944 | 184.92 | 32.114 | 190.12 | 32.106 |
| 7 | 182.25 | 31.496 | 185.58 | 31.687 | 187.10 | 31.697 |
| 8 | 183.16 | 31.442 | 186.34 | 31.635 | 187.84 | 31.646 |
| 9 | 196.03 | 33.039 | 198.82 | 33.467 | 194.76 | 33.309 |
| 10 | 182.69 | 31.720 | 186.49 | 31.817 | 186.89 | 31.900 |
| 11 | 180.67 | 31.467 | 184.33 | 31.747 | 186.24 | 31.774 |
| 12 | 177.57 | 31.520 | 182.11 | 31.817 | 183.45 | 31.839 |
| 13 | 178.54 | 31.692 | 182.87 | 31.981 | 184.50 | 31.919 |
| 14 | 181.33 | 31.343 | 187.53 | 31.697 | 187.94 | 31.520 |
| 15 | 184.61 | 31.608 | 187.69 | 31.804 | 189.34 | 31.748 |
| 16 | 192.43 | 32.006 | 195.33 | 32.240 | 194.42 | 32.112 |
| 17 | 179.03 | 31.630 | 183.66 | 31.874 | 184.40 | 31.838 |
| 18 | 178.99 | 31.442 | 183.95 | 31.714 | 185.01 | 31.680 |
| 19 | 190.68 | 31.860 | 193.91 | 32.065 | 194.40 | 32.023 |
| 20 | 177.14 | 31.310 | 181.40 | 31.589 | 183.10 | 31.632 |
| 21 | 184.68 | 31.583 | 188.14 | 31.721 | 189.02 | 31.670 |
| 22 | 197.84 | 32.295 | 200.24 | 32.682 | 197.32 | 32.754 |
| 23 | 182.94 | 32.415 | 186.80 | 32.540 | 185.01 | 32.369 |

While this study used a methanol reference for ^13^C to best replicate experimental conditions, using TMS as a reference can reduce the MAE of certain methods (FBS 1 to 1.16 ppm) but it raises the MAEs of other methods, including other top FBS that were still able to predict experimental values as shown by having the best *r^2^* and standard deviations values of all FBS studied. Changing the reference compound could be used to decrease the MAE of NMR shift predictions, but in this study, we chose to keep the reference compound consistent with the experimental data. Since using TMS does not show consistent improvement, it is possible that modifying the reference is not inherently improving the prediction but is rather a way to modify some of the data to adjust for a large MSE that results from systematic error in the computation, but these low MAEs by themselves are not sufficient as they do not indicate whether the ordering of shifts is correct.

Table S5: Statistical analysis of top NMR FBS, comparing the effect of the reference compound.

| Reference | NMR FBS # | MAE | MAPE | MSE | *r*^2^ | Stdev | RMSE |
| --- | --- | --- | --- | --- | --- | --- | --- |
| Methanol | 1 | 1.51 | 2.17 | -1.22 | 0.995 | 1.39 | **1.82** |
|  | 7 | 1.85 | 2.61 | -0.49 | 0.996 | 2.19 | **2.20** |
|  | 8 | 1.87 | 2.60 | -0.29 | 0.996 | 2.26 | **2.23** |
|  | 16 | 1.89 | 2.63 | -1.79 | 0.996 | 1.19 | **2.14** |
|  | 19 | 1.72 | 2.51 | -1.52 | 0.996 | 1.43 | **2.07** |
|  | 23 | 1.79 | 2.53 | -1.38 | 0.993 | 1.56 | **2.06** |
| TMS | 1 | 2.30 | 2.87 | 2.30 | 0.995 | 1.39 | 2.67 |
|  | 7 | 2.95 | 3.45 | 2.84 | 0.996 | 2.19 | 3.56 |
|  | 8 | 3.01 | 3.53 | 2.89 | 0.996 | 2.26 | 3.64 |
|  | 16 | 1.26 | 1.56 | 1.11 | 0.996 | 1.19 | 1.61 |
|  | 19 | 1.74 | 2.09 | 1.71 | 0.996 | 1.43 | 2.21 |
|  | 23 | 2.54 | 3.23 | 2.48 | 0.993 | 1.56 | 2.91 |
| Acetone | 1 | 1.16 | 1.55 | 0.33 | 0.995 | 1.39 | 1.40 |
|  | 7 | 4.36 | 5.28 | 4.36 | 0.996 | 2.19 | 4.86 |
|  | 8 | 4.39 | 5.30 | 4.39 | 0.996 | 2.26 | 4.91 |
|  | 16 | 0.98 | 1.29 | 0.20 | 0.996 | 1.19 | 1.18 |
|  | 19 | 2.20 | 2.68 | 2.20 | 0.996 | 1.43 | 2.61 |
|  | 23 | 1.21 | 1.60 | 0.69 | 0.993 | 1.56 | 1.67 |

# **Section S5: Comparison of all NMR FBS combinations with implicit solvation during both steps**


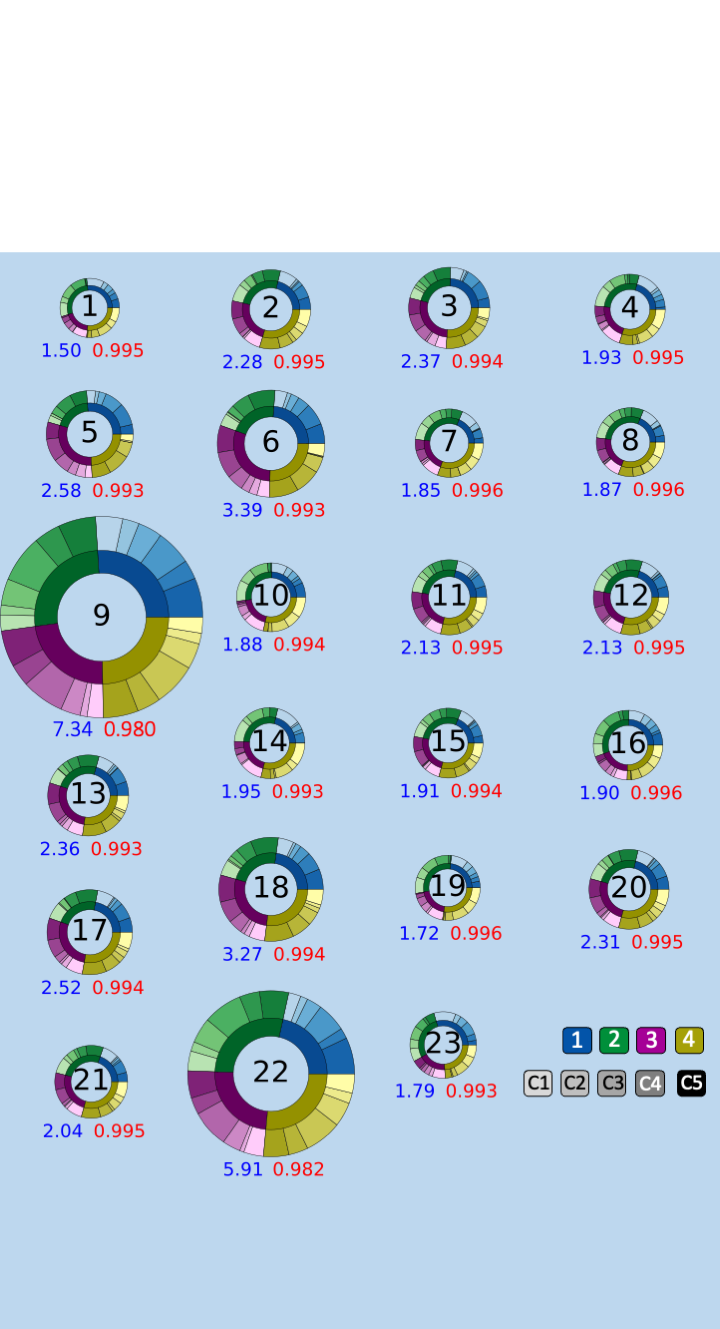


Figure S2: Mean Absolute Error (MAE) for ^13^C NMR Data for NMR FBS 1-21 that were computed in the presence of implicit solvent for both the geometry optimization and NMR SP calculation. The MAEs are with respect to experimental values and are displayed per NMR FBS (denoted by M#) per compound per carbon.

Table S6: The MAE, MAPE, MSE, *r^2^*, standard deviation, and RMSD values for the NMR calculation methods from Figure 7, where values for compound 5 are not considered in the averaged total statistics. For each method, the values are presented overall as well as per carbon and per compound. The total values are an analysis of all predicted shift values for a given method across all compounds and atoms.

|  | Per Compound | | | | | | | Per Carbon Atom | | | | | | |
| --- | --- | --- | --- | --- | --- | --- | --- | --- | --- | --- | --- | --- | --- | --- |
| NMR FBS # | Comp# | MAE (ppm) | MAPE(%) | MSE (ppm) | *r*^2^ | SD (ppm) | RMSD (ppm) | C# | MAE (ppm) | MAPE(%) | MSE (ppm) | *r*^2^ | SD (ppm) | RMSD (ppm) |
| 1 | 1 | 1.61 | 2.28 | -1.18 | 0.993 | 1.53 | 1.83 | 1 | 0.65 | 0.59 | -0.55 | 0.256 | 0.88 | 0.94 |
|  | 2 | 1.70 | 2.42 | -1.70 | 0.997 | 1.33 | 2.09 | 2 | 0.83 | 1.03 | 0.77 | 0.964 | 0.63 | 0.94 |
|  | 3 | 1.11 | 1.63 | -0.72 | 0.996 | 1.37 | 1.44 | 3 | 1.69 | 1.97 | -1.69 | 0.939 | 0.93 | 1.87 |
|  | 4 | 1.61 | 2.35 | -1.27 | 0.997 | 1.50 | 1.87 | 4 | 1.93 | 2.58 | -1.93 | 0.093 | 0.96 | 2.10 |
|  | 5 | 2.14 | 3.06 | -0.05 | 0.978 | 2.73 | 2.50 | 5 | 0.93 | 1.43 | -0.90 | 0.358 | 0.68 | 1.07 |
|  | **TOTAL** | 1.51 | 2.17 | -1.22 | 0.995 | 1.39 | 1.82 | 6 | 3.00 | 5.41 | -3.00 | 0.998 | 0.29 | 3.01 |
| 16 | 1 | 1.86 | 2.57 | -1.66 | 0.995 | 1.36 | 2.07 | 1 | 1.56 | 1.41 | -1.56 | 0.229 | 0.79 | 1.70 |
|  | 2 | 2.34 | 3.19 | -2.34 | 0.997 | 1.08 | 2.54 | 2 | 0.51 | 0.62 | 0.09 | 0.961 | 0.64 | 0.56 |
|  | 3 | 1.45 | 2.02 | -1.30 | 0.997 | 1.12 | 1.66 | 3 | 2.27 | 2.64 | -2.27 | 0.937 | 0.97 | 2.42 |
|  | 4 | 1.92 | 2.74 | -1.87 | 0.997 | 1.27 | 2.20 | 4 | 2.33 | 3.11 | -2.33 | 0.171 | 1.03 | 2.49 |
|  | 5 | 2.03 | 2.82 | -0.54 | 0.980 | 2.58 | 2.41 | 5 | 1.73 | 2.67 | -1.73 | 0.257 | 0.72 | 1.84 |
|  | **TOTAL** | 1.89 | 2.63 | -1.79 | 0.996 | 1.19 | 2.14 | 6 | 2.96 | 5.33 | -2.96 | 0.999 | 0.27 | 2.97 |
| 19 | 1 | 1.59 | 2.31 | -1.27 | 0.995 | 1.41 | 1.81 | 1 | 0.51 | 0.46 | -0.40 | 0.294 | 0.68 | 0.72 |
|  | 2 | 2.04 | 2.94 | -2.04 | 0.998 | 1.39 | 2.40 | 2 | 0.63 | 0.78 | 0.44 | 0.966 | 0.61 | 0.69 |
|  | 3 | 1.35 | 1.99 | -1.06 | 0.996 | 1.44 | 1.69 | 3 | 1.80 | 2.09 | -1.80 | 0.963 | 0.83 | 1.94 |
|  | 4 | 1.89 | 2.80 | -1.71 | 0.998 | 1.65 | 2.28 | 4 | 2.36 | 3.16 | -2.36 | 0.241 | 1.02 | 2.52 |
|  | 5 | 2.09 | 3.04 | -0.25 | 0.978 | 2.74 | 2.51 | 5 | 1.67 | 2.58 | -1.67 | 0.082 | 0.94 | 1.86 |
|  | **TOTAL** | 1.72 | 2.51 | -1.52 | 0.996 | 1.43 | 2.07 | 6 | 3.32 | 5.99 | -3.32 | 1.000 | 0.09 | 3.33 |
| 23 | 1 | 2.12 | 3.09 | -0.70 | 0.985 | 2.60 | 2.47 | 1 | 1.54 | 1.40 | -1.54 | 0.005 | 0.45 | 1.59 |
|  | 2 | 2.05 | 2.79 | -2.05 | 0.999 | 0.80 | 2.17 | 2 | 0.66 | 0.83 | 0.37 | 0.967 | 0.81 | 0.79 |
|  | 3 | 1.28 | 1.79 | -1.14 | 0.997 | 1.07 | 1.50 | 3 | 1.73 | 2.06 | -0.28 | 0.801 | 2.21 | 1.94 |
|  | 4 | 1.72 | 2.45 | -1.63 | 0.997 | 1.17 | 1.95 | 4 | 2.09 | 2.79 | -2.09 | 0.006 | 0.84 | 2.21 |
|  | 5 | 2.50 | 3.34 | -0.21 | 0.972 | 3.09 | 2.83 | 5 | 1.54 | 2.36 | -1.54 | 0.107 | 0.67 | 1.64 |
|  | **TOTAL** | 1.79 | 2.53 | -1.38 | 0.993 | 1.56 | 2.06 | 6 | 3.19 | 5.73 | -3.19 | 0.901 | 0.94 | 3.29 |
| 7 | 1 | 1.38 | 2.06 | -0.34 | 0.995 | 1.99 | 1.85 | 1 | 2.14 | 1.94 | 2.14 | 0.244 | 0.72 | 2.23 |
|  | 2 | 2.19 | 3.05 | -1.00 | 0.997 | 2.30 | 2.33 | 2 | 1.92 | 2.35 | 1.92 | 0.975 | 0.49 | 1.97 |
|  | 3 | 1.56 | 2.17 | 0.04 | 0.996 | 2.25 | 2.05 | 3 | 0.37 | 0.43 | -0.31 | 0.967 | 0.65 | 0.64 |
|  | 4 | 2.26 | 3.15 | -0.64 | 0.997 | 2.66 | 2.52 | 4 | 1.40 | 1.87 | -1.40 | 0.392 | 1.26 | 1.78 |
|  | 5 | 2.31 | 3.46 | 0.68 | 0.978 | 3.00 | 2.82 | 5 | 1.63 | 2.51 | -1.63 | 0.047 | 0.98 | 1.84 |
|  | **TOTAL** | 1.85 | 2.61 | -0.49 | 0.996 | 2.19 | 2.20 | 6 | 3.64 | 6.56 | -3.64 | 0.999 | 0.06 | 3.64 |
| 8 | 1 | 1.41 | 2.06 | -0.16 | 0.995 | 2.04 | 1.86 | 1 | 2.48 | 2.24 | 2.48 | 0.240 | 0.73 | 2.55 |
|  | 2 | 2.18 | 3.00 | -0.81 | 0.997 | 2.38 | 2.31 | 2 | 2.14 | 2.62 | 2.14 | 0.975 | 0.49 | 2.19 |
|  | 3 | 1.60 | 2.17 | 0.25 | 0.996 | 2.32 | 2.14 | 3 | 0.40 | 0.46 | -0.16 | 0.967 | 0.64 | 0.58 |
|  | 4 | 2.30 | 3.15 | -0.44 | 0.997 | 2.74 | 2.54 | 4 | 1.25 | 1.67 | -1.24 | 0.395 | 1.27 | 1.65 |
|  | 5 | 2.45 | 3.64 | 0.85 | 0.978 | 3.05 | 2.91 | 5 | 1.42 | 2.18 | -1.42 | 0.045 | 1.00 | 1.66 |
|  | **TOTAL** | 1.87 | 2.60 | -0.29 | 0.996 | 2.26 | 2.23 | 6 | 3.56 | 6.41 | -3.56 | 0.999 | 0.06 | 3.56 |

**Section S6: Effect of geometry FBS**

# In literature, it is recommended that NMR FBS 1, 15, and 16 be combined with specific geometry optimization FBS to yield accurate NMR shift predictions. However, the higher errors exhibited when using these geometry FBSs suggests that our use of B3LYP/6-31+G(d,p) for geometry optimizations both allows us to hold the geometry FBS constant to more accurately study the effect of the NMR FBS and solvation models, and perform better than other FBSs recommended in literature for NMR predictions. The results below used implicit solvation for both the optimization and NMR calculations, focusing on charge-neutral compound 1.

# Figure S3: MAE’s (ppm) for 13C NMR predictions for compound 1 using NMR FBS 1, 15, and 16 combined with different geometry optimization FBS suggested in literature. Note this data considers the E0 pucker state for Compound 1.

**Section S7: 1H NMR Shift predictions**

# Table S7: Chemical Shift predictions for 1H using implicit solvation during both geometry optimizations and NMR calculations for the top 6 methods as compared with literature values from Ishii et al.

| Compound | Method | H1 | H2 | H3’a | H3’b | H4a | H4b | OCH_3_ |
| --- | --- | --- | --- | --- | --- | --- | --- | --- |
| 1 | 1 | 4.968 | 3.723 | 3.935 | 3.703 | 4.073 | 3.938 | 3.488 |
|  | 7 | 4.934 | 3.625 | 3.906 | 3.569 | 4.019 | 3.928 | 3.395 |
|  | 8 | 4.931 | 3.620 | 3.910 | 3.562 | 4.024 | 3.935 | 3.397 |
|  | 16 | 4.944 | 3.712 | 3.871 | 3.700 | 4.078 | 3.869 | 3.455 |
|  | 19 | 4.970 | 3.739 | 3.874 | 3.648 | 4.070 | 3.968 | 3.486 |
|  | 23 | 4.923 | 3.698 | 3.810 | 3.659 | 3.977 | 3.840 | 3.385 |
|  | Lit | 4.968 | 3.936 | 3.637 | 3.637 | 3.891 | 4.044 | 3.441 |
| 2 | 1 | 4.721 | 4.076 | 3.445 | 3.616 | 3.438 | 3.855 | 3.365 |
|  | 7 | 4.768 | 4.139 | 3.349 | 3.635 | 3.537 | 3.941 | 3.318 |
|  | 8 | 4.767 | 4.144 | 3.346 | 3.637 | 3.533 | 3.949 | 3.320 |
|  | 16 | 4.700 | 4.045 | 3.428 | 3.578 | 3.475 | 3.773 | 3.326 |
|  | 19 | 4.790 | 4.212 | 3.482 | 3.659 | 3.580 | 3.860 | 3.369 |
|  | 23 | 4.669 | 3.975 | 3.355 | 3.505 | 3.451 | 3.714 | 3.261 |
|  | Lit | 4.902 | 4.019 | 3.655 | 3.608 | 3.847 | 3.800 | 3.339 |
| 3 | 1 | 4.749 | 4.020 | 3.519 | 3.673 | 3.759 | 3.939 | 3.383 |
|  | 7 | 4.731 | 3.979 | 3.395 | 3.665 | 3.714 | 3.906 | 3.299 |
|  | 8 | 4.730 | 3.986 | 3.397 | 3.669 | 3.716 | 3.914 | 3.301 |
|  | 16 | 4.728 | 3.981 | 3.490 | 3.634 | 3.786 | 3.862 | 3.343 |
|  | 19 | 4.779 | 4.060 | 3.524 | 3.688 | 3.814 | 3.926 | 3.369 |
|  | 23 | 4.705 | 3.922 | 3.406 | 3.567 | 3.714 | 3.805 | 3.280 |
|  | Lit | 4.889 | 4.005 | 3.581 | 3.647 | 3.870 | 3.813 | 3.333 |
| 4 | 1 | 4.722 | 4.246 | 3.528 | 3.660 | 3.576 | 3.894 | 3.376 |
|  | 7 | 4.696 | 4.189 | 3.364 | 3.622 | 3.563 | 3.902 | 3.290 |
|  | 8 | 4.695 | 4.195 | 3.362 | 3.625 | 3.561 | 3.912 | 3.293 |
|  | 16 | 4.700 | 4.210 | 3.507 | 3.620 | 3.609 | 3.810 | 3.337 |
|  | 19 | 4.739 | 4.308 | 3.533 | 3.656 | 3.644 | 3.858 | 3.360 |
|  | 23 | 4.680 | 4.140 | 3.441 | 3.554 | 3.569 | 3.750 | 3.274 |
|  | Lit | 4.899 | 4.005 | 3.584 | 3.647 | 3.838 | 3.805 | 3.334 |

**Section S8: Effect of solvation model combination on shift predictions**

Table S8: Predicted ^13^C chemical shifts for the top six NMR FBS: 1, 23, 16, 19, 7, and 8.

| Solvation Model | | | NMR  FBS | | Comp | | Chemical Shift by Carbon # | | | | | | | Comp’s  MAE | | FBS’s  MAE | |
| --- | --- | --- | --- | --- | --- | --- | --- | --- | --- | --- | --- | --- | --- | --- | --- | --- | --- |
| Geo | NMR | # | | # | | 1 | | 3 | 2 | 4 | 5 | 6 | (ppm) | | (ppm) | |  |
| Gas | Gas | 1 | | 1 | | 108.55 | | 78.97 | 78.90 | 72.77 | 64.84 | 52.61 | 1.81 | | 2.49 | |  |
| Gas | SCRF | 1 | | 1 | | 108.53 | | 78.76 | 79.62 | 73.17 | 64.47 | 52.73 | 1.50 | | 2.37 | |  |
| SCRF | Gas | 1 | | 1 | | 108.45 | | 78.79 | 78.51 | 72.62 | 63.68 | 53.27 | 1.76 | | 1.65 | |  |
| SCRF | SCRF | 1 | | 1 | | 108.38 | | 78.59 | 79.25 | 73.04 | 63.26 | 53.35 | 1.60 | | 1.50 | |  |
| Gas | Gas | 1 | | 2 | | 108.32 | | 85.92 | 84.26 | 77.73 | 66.85 | 50.03 | 2.78 | |  |  |  |
| Gas | SCRF | 1 | | 2 | | 107.08 | | 85.83 | 84.03 | 77.21 | 66.50 | 50.30 | 2.82 | |  |  |  |
| SCRF | Gas | 1 | | 2 | | 111.21 | | 83.45 | 83.28 | 72.47 | 65.56 | 51.84 | 1.70 | |  |  |  |
| SCRF | SCRF | 1 | | 2 | | 110.41 | | 83.30 | 83.92 | 72.33 | 64.20 | 52.24 | 1.70 | |  |  |  |
| Gas | Gas | 1 | | 3 | | 109.15 | | 86.31 | 84.82 | 77.05 | 66.98 | 50.66 | 2.47 | |  |  |  |
| Gas | SCRF | 1 | | 3 | | 108.45 | | 85.85 | 84.92 | 76.95 | 66.43 | 50.96 | 2.33 | |  |  |  |
| SCRF | Gas | 1 | | 3 | | 110.56 | | 84.75 | 85.33 | 74.09 | 65.86 | 51.80 | 1.40 | |  |  |  |
| SCRF | SCRF | 1 | | 3 | | 109.90 | | 84.36 | 85.71 | 73.92 | 65.20 | 52.17 | 1.11 | |  |  |  |
| Gas | Gas | 1 | | 4 | | 107.68 | | 87.30 | 84.40 | 76.68 | 66.82 | 50.11 | 2.91 | |  |  |  |
| Gas | SCRF | 1 | | 4 | | 106.76 | | 86.90 | 84.25 | 76.68 | 66.11 | 50.49 | 2.84 | |  |  |  |
| SCRF | Gas | 1 | | 4 | | 111.29 | | 84.40 | 85.06 | 72.26 | 64.51 | 51.86 | 1.72 | |  |  |  |
| SCRF | SCRF | 1 | | 4 | | 110.48 | | 84.24 | 85.39 | 72.16 | 63.64 | 52.22 | 1.61 | |  |  |  |
| Gas | Gas | 23 | | 1 | | 107.35 | | 78.79 | 80.46 | 72.62 | 64.27 | 52.78 | 1.74 | | 2.16 | |  |
| Gas | SCRF | 23 | | 1 | | 107.26 | | 78.56 | 81.19 | 72.98 | 63.93 | 52.85 | 1.81 | | 2.13 | |  |
| SCRF | Gas | 23 | | 1 | | 109.24 | | 78.67 | 82.25 | 71.98 | 63.36 | 51.88 | 2.10 | | 1.67 | |  |
| SCRF | SCRF | 23 | | 1 | | 109.23 | | 78.68 | 82.98 | 72.59 | 63.07 | 52.19 | 2.12 | | 1.79 | |  |
| Gas | Gas | 23 | | 2 | | 107.36 | | 84.71 | 85.32 | 77.65 | 66.02 | 50.36 | 2.36 | |  |  |  |
| Gas | SCRF | 23 | | 2 | | 106.13 | | 84.52 | 85.09 | 77.15 | 65.58 | 50.61 | 2.37 | |  |  |  |
| SCRF | Gas | 23 | | 2 | | 109.57 | | 83.05 | 84.10 | 72.43 | 64.66 | 51.98 | 1.80 | |  |  |  |
| SCRF | SCRF | 23 | | 2 | | 108.73 | | 82.83 | 84.68 | 72.29 | 63.38 | 52.37 | 2.05 | |  |  |  |
| Gas | Gas | 23 | | 3 | | 108.06 | | 85.16 | 85.63 | 76.88 | 66.04 | 50.92 | 2.09 | |  |  |  |
| Gas | SCRF | 23 | | 3 | | 107.32 | | 84.66 | 85.70 | 76.75 | 65.47 | 51.19 | 1.96 | |  |  |  |
| SCRF | Gas | 23 | | 3 | | 109.08 | | 84.13 | 85.94 | 73.96 | 65.02 | 51.95 | 1.21 | |  |  |  |
| SCRF | SCRF | 23 | | 3 | | 108.36 | | 83.68 | 86.27 | 73.78 | 64.34 | 52.31 | 1.28 | |  |  |  |
| Gas | Gas | 23 | | 4 | | 106.80 | | 85.86 | 85.39 | 76.69 | 65.86 | 50.45 | 2.43 | |  |  |  |
| Gas | SCRF | 23 | | 4 | | 105.86 | | 85.41 | 85.23 | 76.64 | 65.13 | 50.80 | 2.36 | |  |  |  |
| SCRF | Gas | 23 | | 4 | | 109.72 | | 83.92 | 85.69 | 72.24 | 63.78 | 52.01 | 1.56 | |  |  |  |
| SCRF | SCRF | 23 | | 4 | | 108.86 | | 83.67 | 85.97 | 72.14 | 62.93 | 52.35 | 1.72 | |  |  |  |
| Gas | Gas | 16 | | 1 | | 107.72 | | 78.32 | 78.46 | 72.50 | 64.05 | 52.71 | 1.87 | | 2.41 | |  |
| Gas | SCRF | 16 | | 1 | | 107.68 | | 78.10 | 79.21 | 72.92 | 63.70 | 52.80 | 1.68 | | 2.31 | |  |
| SCRF | Gas | 16 | | 1 | | 107.60 | | 78.11 | 78.03 | 72.34 | 62.92 | 53.38 | 2.02 | | 1.84 | |  |
| SCRF | SCRF | 16 | | 1 | | 107.50 | | 77.89 | 78.80 | 72.78 | 62.53 | 53.42 | 1.86 | | 1.90 | |  |
| Gas | Gas | 16 | | 2 | | 107.48 | | 85.11 | 83.61 | 77.03 | 65.98 | 50.09 | 2.63 | |  |  |  |
| Gas | SCRF | 16 | | 2 | | 106.30 | | 85.00 | 83.40 | 76.54 | 65.60 | 50.36 | 2.65 | |  |  |  |
| SCRF | Gas | 16 | | 2 | | 110.05 | | 82.74 | 82.64 | 71.88 | 64.58 | 51.84 | 2.14 | |  |  |  |
| SCRF | SCRF | 16 | | 2 | | 109.29 | | 82.58 | 83.31 | 71.81 | 63.26 | 52.26 | 2.34 | |  |  |  |
| Gas | Gas | 16 | | 3 | | 108.29 | | 85.59 | 84.13 | 76.58 | 66.18 | 50.70 | 2.39 | |  |  |  |
| Gas | SCRF | 16 | | 3 | | 107.61 | | 85.12 | 84.23 | 76.48 | 65.62 | 50.99 | 2.25 | |  |  |  |
| SCRF | Gas | 16 | | 3 | | 109.53 | | 84.09 | 84.67 | 73.64 | 65.05 | 51.82 | 1.41 | |  |  |  |
| SCRF | SCRF | 16 | | 3 | | 108.88 | | 83.67 | 85.07 | 73.51 | 64.38 | 52.20 | 1.45 | |  |  |  |
| Gas | Gas | 16 | | 4 | | 106.89 | | 86.47 | 83.70 | 76.09 | 65.93 | 50.19 | 2.76 | |  |  |  |
| Gas | SCRF | 16 | | 4 | | 106.00 | | 86.07 | 83.56 | 76.08 | 65.20 | 50.56 | 2.68 | |  |  |  |
| SCRF | Gas | 16 | | 4 | | 110.21 | | 83.77 | 84.41 | 71.77 | 63.58 | 51.88 | 1.80 | |  |  |  |
| SCRF | SCRF | 16 | | 4 | | 109.42 | | 83.58 | 84.77 | 71.73 | 62.74 | 52.25 | 1.93 | |  |  |  |
| Gas | Gas | 19 | | 1 | | 109.63 | | 79.24 | 79.53 | 72.94 | 64.96 | 52.83 | 1.52 | | 2.40 | |  |
| Gas | SCRF | 19 | | 1 | | 109.15 | | 78.59 | 79.70 | 73.15 | 64.09 | 52.70 | 1.35 | | 2.24 | |  |
| SCRF | Gas | 19 | | 1 | | 109.42 | | 78.90 | 79.04 | 72.70 | 63.75 | 53.50 | 1.47 | | 1.74 | |  |
| SCRF | SCRF | 19 | | 1 | | 108.82 | | 78.25 | 79.21 | 72.87 | 62.80 | 53.32 | 1.59 | | 1.72 | |  |
| Gas | Gas | 19 | | 2 | | 109.66 | | 85.66 | 84.27 | 77.79 | 67.35 | 49.62 | 2.68 | |  |  |  |
| Gas | SCRF | 19 | | 2 | | 107.78 | | 84.96 | 83.60 | 76.93 | 66.21 | 49.84 | 2.62 | |  |  |  |
| SCRF | Gas | 19 | | 2 | | 111.90 | | 83.57 | 83.94 | 72.18 | 65.30 | 51.26 | 1.85 | |  |  |  |
| SCRF | SCRF | 19 | | 2 | | 110.47 | | 83.04 | 84.03 | 71.87 | 63.17 | 51.80 | 2.03 | |  |  |  |
| Gas | Gas | 19 | | 3 | | 110.02 | | 86.56 | 84.92 | 77.08 | 67.68 | 50.26 | 2.54 | |  |  |  |
| Gas | SCRF | 19 | | 3 | | 108.82 | | 85.52 | 84.50 | 76.69 | 66.46 | 50.53 | 2.32 | |  |  |  |
| SCRF | Gas | 19 | | 3 | | 111.17 | | 84.94 | 85.57 | 73.64 | 66.06 | 51.30 | 1.68 | |  |  |  |
| SCRF | SCRF | 19 | | 3 | | 109.97 | | 84.11 | 85.34 | 73.35 | 64.67 | 51.77 | 1.35 | |  |  |  |
| Gas | Gas | 19 | | 4 | | 108.79 | | 87.09 | 84.26 | 76.62 | 67.45 | 49.81 | 2.86 | |  |  |  |
| Gas | SCRF | 19 | | 4 | | 107.32 | | 86.05 | 83.66 | 76.21 | 66.06 | 50.08 | 2.68 | |  |  |  |
| SCRF | Gas | 19 | | 4 | | 111.89 | | 84.28 | 85.51 | 71.81 | 64.16 | 51.26 | 1.96 | |  |  |  |
| SCRF | SCRF | 19 | | 4 | | 110.49 | | 83.75 | 85.26 | 71.62 | 62.55 | 51.79 | 1.89 | |  |  |  |
| Gas | Gas | 7 | | 1 | | 112.15 | | 80.08 | 80.54 | 74.29 | 65.30 | 52.63 | 1.74 | | 2.72 | |  |
| Gas | SCRF | 7 | | 1 | | 111.70 | | 79.48 | 80.72 | 74.40 | 64.45 | 52.43 | 1.49 | | 2.29 | |  |
| SCRF | Gas | 7 | | 1 | | 111.88 | | 79.80 | 80.04 | 74.08 | 63.86 | 53.29 | 1.38 | | 1.99 | |  |
| SCRF | SCRF | 7 | | 1 | | 111.33 | | 79.18 | 80.20 | 74.17 | 62.93 | 53.05 | 1.38 | | 1.85 | |  |
| Gas | Gas | 7 | | 2 | | 112.13 | | 87.39 | 86.34 | 79.13 | 67.23 | 49.17 | 2.96 | |  |  |  |
| Gas | SCRF | 7 | | 2 | | 110.27 | | 86.69 | 85.70 | 78.31 | 66.29 | 49.27 | 2.48 | |  |  |  |
| SCRF | Gas | 7 | | 2 | | 114.32 | | 85.19 | 85.37 | 72.89 | 65.02 | 51.12 | 2.24 | |  |  |  |
| SCRF | SCRF | 7 | | 2 | | 112.96 | | 84.71 | 85.64 | 72.61 | 63.15 | 51.48 | 2.19 | |  |  |  |
| Gas | Gas | 7 | | 3 | | 112.63 | | 88.28 | 86.84 | 78.42 | 67.80 | 49.94 | 3.18 | |  |  |  |
| Gas | SCRF | 7 | | 3 | | 111.45 | | 87.27 | 86.48 | 78.01 | 66.69 | 50.07 | 2.60 | |  |  |  |
| SCRF | Gas | 7 | | 3 | | 113.68 | | 86.55 | 87.12 | 74.68 | 65.97 | 51.13 | 1.96 | |  |  |  |
| SCRF | SCRF | 7 | | 3 | | 112.52 | | 85.72 | 87.05 | 74.38 | 64.72 | 51.42 | 1.56 | |  |  |  |
| Gas | Gas | 7 | | 4 | | 111.25 | | 88.94 | 86.51 | 78.07 | 67.44 | 49.37 | 3.02 | |  |  |  |
| Gas | SCRF | 7 | | 4 | | 109.78 | | 87.96 | 85.92 | 77.68 | 66.22 | 49.51 | 2.58 | |  |  |  |
| SCRF | Gas | 7 | | 4 | | 114.46 | | 85.98 | 86.99 | 72.57 | 63.91 | 51.15 | 2.37 | |  |  |  |
| SCRF | SCRF | 7 | | 4 | | 113.10 | | 85.46 | 86.90 | 72.39 | 62.54 | 51.47 | 2.26 | |  |  |  |
| Gas | Gas | 8 | | 1 | | 112.45 | | 80.27 | 80.62 | 74.46 | 65.53 | 52.70 | 1.88 | | 2.86 | |  |
| Gas | SCRF | 8 | | 1 | | 112.00 | | 79.67 | 80.80 | 74.56 | 64.67 | 52.50 | 1.64 | | 2.38 | |  |
| SCRF | Gas | 8 | | 1 | | 112.19 | | 79.99 | 80.13 | 74.25 | 64.08 | 53.37 | 1.39 | | 2.04 | |  |
| SCRF | SCRF | 8 | | 1 | | 111.64 | | 79.37 | 80.28 | 74.34 | 63.14 | 53.14 | 1.41 | | 1.87 | |  |
| Gas | Gas | 8 | | 2 | | 112.40 | | 87.67 | 86.48 | 79.30 | 67.47 | 49.22 | 3.09 | |  |  |  |
| Gas | SCRF | 8 | | 2 | | 110.53 | | 86.97 | 85.84 | 78.47 | 66.53 | 49.32 | 2.52 | |  |  |  |
| SCRF | Gas | 8 | | 2 | | 114.67 | | 85.41 | 85.52 | 73.05 | 65.23 | 51.19 | 2.24 | |  |  |  |
| SCRF | SCRF | 8 | | 2 | | 113.30 | | 84.94 | 85.80 | 72.77 | 63.35 | 51.56 | 2.18 | |  |  |  |
| Gas | Gas | 8 | | 3 | | 112.91 | | 88.56 | 87.00 | 78.58 | 68.08 | 49.99 | 3.31 | |  |  |  |
| Gas | SCRF | 8 | | 3 | | 111.74 | | 87.54 | 86.63 | 78.18 | 66.96 | 50.12 | 2.73 | |  |  |  |
| SCRF | Gas | 8 | | 3 | | 114.01 | | 86.79 | 87.29 | 74.84 | 66.21 | 51.20 | 2.09 | |  |  |  |
| SCRF | SCRF | 8 | | 3 | | 112.86 | | 85.96 | 87.22 | 74.54 | 64.96 | 51.50 | 1.60 | |  |  |  |
| Gas | Gas | 8 | | 4 | | 111.52 | | 89.25 | 86.65 | 78.23 | 67.69 | 49.41 | 3.15 | |  |  |  |
| Gas | SCRF | 8 | | 4 | | 110.05 | | 88.26 | 86.06 | 77.84 | 66.47 | 49.56 | 2.62 | |  |  |  |
| SCRF | Gas | 8 | | 4 | | 114.81 | | 86.19 | 87.15 | 72.73 | 64.11 | 51.22 | 2.43 | |  |  |  |
| SCRF | SCRF | 8 | | 4 | | 113.45 | | 85.68 | 87.07 | 72.55 | 62.74 | 51.55 | 2.30 | |  |  |  |

Using implicit solvation for both combinations yields the most accurate results for all methods, and while it is not the lowest MAE option for NMR FBS 9 and 16, the gas-phase NMR predictions do not consistently or clearly predict the shift ordering, some of which is also reflected in the statistical analysis shown below. Blue highlighted values show where the top performing method was the SCRF/SCRF combination for both steps, whereas the orange highlights show where the top performing method is the SCRF/Gas combination. The purely implicit solvation models consistently have the best *r^2^* and SD values, and almost always have the best RMSD values even if the MAE/MAPE suggest a gas-phase NMR calculation.

Table S9: Statistical analysis of the effect of four solvation combinations on the top six NMR FBS.

| Solvation Model | |  |  |  |  |  |  |  |
| --- | --- | --- | --- | --- | --- | --- | --- | --- |
| Geo | NMR | NMR  FBS# | MAE  (ppm) | MAPE  (%) | MSE  (ppm) | *r*^2^ | SD  (ppm) | RMSD  (ppm) |
| Gas | Gas | 1 | 2.49 | 3.48 | -0.55 | 0.977 | 2.76 | 2.76 |
| Gas | SCRF | 1 | 2.37 | 3.24 | -0.74 | 0.978 | 2.64 | 2.68 |
| SCRF | Gas | 1 | 1.65 | 2.33 | -1.08 | 0.993 | 1.70 | 1.98 |
| SCRF | SCRF | 1 | 1.50 | 2.16 | -1.21 | 0.995 | 1.39 | 1.82 |
| Gas | Gas | 7 | 2.72 | 3.89 | 0.88 | 0.978 | 3.24 | 3.29 |
| Gas | SCRF | 7 | 2.29 | 3.33 | 0.25 | 0.980 | 2.94 | 2.89 |
| SCRF | Gas | 7 | 1.99 | 2.69 | 0.01 | 0.994 | 2.52 | 2.47 |
| SCRF | SCRF | 7 | 1.85 | 2.61 | -0.49 | 0.996 | 2.19 | 2.20 |
| Gas | Gas | 8 | 2.86 | 4.05 | 1.07 | 0.977 | 3.31 | 3.41 |
| Gas | SCRF | 8 | 2.38 | 3.45 | 0.44 | 0.980 | 3.01 | 2.98 |
| SCRF | Gas | 8 | 2.04 | 2.71 | 0.20 | 0.994 | 2.60 | 2.55 |
| SCRF | SCRF | 8 | 1.87 | 2.60 | -0.29 | 0.996 | 2.26 | 2.23 |
| Gas | Gas | 23 | 2.16 | 2.96 | -0.81 | 0.981 | 2.43 | 2.52 |
| Gas | SCRF | 23 | 2.13 | 2.83 | -1.03 | 0.982 | 2.39 | 2.56 |
| SCRF | Gas | 23 | 1.67 | 2.40 | -1.26 | 0.993 | 1.62 | 2.02 |
| SCRF | SCRF | 23 | 1.79 | 2.53 | -1.38 | 0.993 | 1.56 | 2.06 |
| Gas | Gas | 16 | 2.41 | 3.28 | -1.12 | 0.980 | 2.56 | 2.75 |
| Gas | SCRF | 16 | 2.31 | 3.08 | -1.32 | 0.981 | 2.46 | 2.74 |
| SCRF | Gas | 16 | 1.84 | 2.59 | -1.68 | 0.994 | 1.47 | 2.21 |
| SCRF | SCRF | 16 | 1.90 | 2.63 | -1.80 | 0.996 | 1.19 | 2.14 |
| Gas | Gas | 19 | 2.40 | 3.47 | -0.28 | 0.977 | 2.83 | 2.78 |
| Gas | SCRF | 19 | 2.24 | 3.13 | -0.92 | 0.981 | 2.53 | 2.64 |
| SCRF | Gas | 19 | 1.74 | 2.48 | -0.99 | 0.993 | 1.87 | 2.08 |
| SCRF | SCRF | 19 | 1.72 | 2.51 | -1.52 | 0.996 | 1.43 | 2.07 |

**Section S9: Calculating shifts using scaling factors**

# Pierens only reported scaling factors for NMR FBS 1-10, so only the top performing FBS 1, 7, 8, and 10 are reported below. Scaling factors were specific to an NMR FBS, specific experimental solvent (i.e. water), and the solvation model used for NMR calculations. We used the scaling factors reported for water as it has a similar dielectric to D_2_O. While all scaling factors were calculated using gas-phase geometry optimizations, our results show that the consideration of solvent during Geometry optimizations greatly improves the predictions. The best MAE reported using Pierens’s scaling factors on compounds 1-4 is 2.51 ppm, which is over 60% worse than this study’s best MAE of 1.50 ppm that resulted from a constant reference correction, a lower MAE than reported by Pierens’s when they applied their scaling factors to a test compound.

# Table S10: Statistical analysis of chemical shifts calculated by applying Pierens’s scaling factors on compounds 1-4. Note this data considers the E0 pucker state for Compound 1.

| Solvation Model | |  |  |  |  |  |  |  |
| --- | --- | --- | --- | --- | --- | --- | --- | --- |
| Geometry | NMR | NMR FBS | MAE  (ppm) | MAPE  (%) | MSE  (ppm) | *r^2^* | Stdev  (ppm) | RMSE  (ppm) |
| Gas | Gas | 1 | 6.70 | 8.90 | -6.70 | 0.970 | 3.10 | 7.35 |
|  |  | 7 | 3.86 | 5.28 | 2.54 | 0.969 | 3.50 | 4.27 |
|  |  | 8 | 3.96 | 5.41 | 2.64 | 0.969 | 3.57 | 4.38 |
|  |  | 10 | 4.98 | 6.47 | 4.95 | 0.974 | 2.88 | 5.70 |
| Gas | Implicit | 1 | 8.77 | 11.70 | -8.77 | 0.971 | 3.03 | 9.26 |
|  |  | 7 | 2.76 | 4.03 | 0.50 | 0.971 | 3.48 | 3.44 |
|  |  | 8 | 2.83 | 4.13 | 0.58 | 0.971 | 3.54 | 3.52 |
|  |  | 10 | 3.78 | 5.03 | 3.11 | 0.975 | 2.85 | 4.17 |
| Implicit | Gas | 1 | 6.73 | 9.07 | -6.73 | 0.989 | 1.92 | 6.98 |
|  |  | 7 | 3.05 | 3.83 | 2.30 | 0.989 | 2.81 | 3.58 |
|  |  | 8 | 3.14 | 3.94 | 2.40 | 0.989 | 2.88 | 3.70 |
|  |  | 10 | 4.73 | 6.06 | 4.73 | 0.987 | 2.21 | 5.20 |
| Implicit | Implicit | 1 | 8.72 | 11.81 | -8.72 | 0.991 | 1.86 | 8.91 |
|  |  | 7 | 2.51 | 3.39 | 0.44 | 0.991 | 2.90 | 2.87 |
|  |  | 8 | 2.57 | 3.45 | 0.53 | 0.991 | 2.97 | 2.95 |
|  |  | 10 | 3.11 | 3.87 | 3.04 | 0.987 | 2.27 | 3.76 |

**Section S10: Optimized Geometry Coordinates**

The optimized geometry coordinates are presented below for compounds 1-5 with both implicit and gas-phase solvation models. Additionally, sums of electronic and thermal free energies are reported. The numbering between an atom’s center number and the naming convention is also reported below. While we reported NMR data for 15 atoms (36 atoms for dimers), we do not NMR present data for the oxygens atoms (^17^O), alcohol protons (^1^H), or boron atoms (^11^B). Charges were specified in the input files and were not assigned to specific atoms.

Table S11: Relationship between atom naming convention and center number in geometry coordinates, with values presented for both monomer compound (1, 2, 5) and dimer compounds that have multiple equivalent atoms (3, 4).

| Naming Convention | Center Number  (monomer) | Center Number  (dimer) |
| --- | --- | --- |
| C1 | 1 | 22 |
| C2 | 2 | 23 |
| C3 | 3 | 24 |
| C4 | 4 | 25 |
| C3' | 5 | 26 |
| OCH_3_ (carbon) | 6 | 27 |
| H1 | 7 | 28 |
| H2 | 8 | 29 |
| H3’a | 9 | 30 |
| H3’b | 10 | 31 |
| H4a | 11 | 32 |
| H4b | 12 | 33 |
| OCH_3_ (hydrogen) | 13 | 34 |
| OCH_3_ (hydrogen) | 14 | 35 |
| OCH_3_ (hydrogen) | 15 | 36 |

#REMARK Compound 1 – Implicit Solvation Geometry

#REMARK E0 Pucker State

#REMARK Sum of electronic and thermal Free Energies= -611.847720

HETATM 1 C C1 1 1.411 0.445 0.140

HETATM 2 C C1 1 0.127 0.919 -0.574

HETATM 3 C C1 1 -1.011 0.044 0.066

HETATM 4 C C1 1 -0.238 -0.915 0.982

HETATM 5 C C1 1 -1.869 -0.658 -0.987

HETATM 6 C C1 1 3.351 -0.885 -0.245

HETATM 7 H C1 1 2.061 1.268 0.464

HETATM 8 H C1 1 0.213 0.729 -1.646

HETATM 9 H C1 1 -1.250 -1.312 -1.608

HETATM 10 H C1 1 -2.331 0.095 -1.641

HETATM 11 H C1 1 -0.769 -1.121 1.912

HETATM 12 H C1 1 -0.015 -1.858 0.470

HETATM 13 H C1 1 3.206 -1.523 0.631

HETATM 14 H C1 1 3.807 -1.466 -1.049

HETATM 15 H C1 1 4.010 -0.047 0.018

HETATM 16 H C1 1 -0.057 2.567 0.482

HETATM 17 H C1 1 -2.128 1.625 0.409

HETATM 18 H C1 1 -3.358 -0.932 0.237

HETATM 19 O C1 1 0.982 -0.227 1.312

HETATM 20 O C1 1 2.100 -0.403 -0.749

HETATM 21 O C1 1 -0.111 2.313 -0.451

HETATM 22 O C1 1 -1.857 0.838 0.908

HETATM 23 O C1 1 -2.866 -1.485 -0.389

#REMARK Compound 1 – Implicit Solvation Geometry

#REMARK 4T3 Pucker State

#REMARK Sum of electronic and thermal Free Energies= -611.849402

HETATM 1 C C1 1 1.399 0.215 0.284

HETATM 2 C C1 1 0.152 0.878 -0.353

HETATM 3 C C1 1 -1.019 -0.057 0.045

HETATM 4 C C1 1 -0.284 -1.389 0.246

HETATM 5 C C1 1 -2.155 -0.069 -0.984

HETATM 6 C C1 1 3.623 -0.448 -0.259

HETATM 7 H C1 1 1.826 0.812 1.099

HETATM 8 H C1 1 0.287 0.868 -1.440

HETATM 9 H C1 1 -1.846 -0.585 -1.900

HETATM 10 H C1 1 -2.419 0.961 -1.237

HETATM 11 H C1 1 -0.800 -2.068 0.927

HETATM 12 H C1 1 -0.114 -1.894 -0.715

HETATM 13 H C1 1 3.533 -1.450 0.171

HETATM 14 H C1 1 4.287 -0.480 -1.124

HETATM 15 H C1 1 4.038 0.236 0.493

HETATM 16 H C1 1 -0.601 2.192 0.840

HETATM 17 H C1 1 -2.449 0.151 1.355

HETATM 18 H C1 1 -3.285 -1.615 -0.480

HETATM 19 O C1 1 0.968 -1.020 0.851

HETATM 20 O C1 1 2.358 0.028 -0.731

HETATM 21 O C1 1 -0.056 2.221 0.034

HETATM 22 O C1 1 -1.520 0.433 1.295

HETATM 23 O C1 1 -3.343 -0.651 -0.431

#REMARK Compound 2 – Implicit Solvation Geometry

#REMARK Sum of electronic and thermal Free Energies= -787.457619

HETATM 1 C C2 1 -1.181 -0.963 -0.261

HETATM 2 C C2 1 -0.111 -0.020 -0.854

HETATM 3 C C2 1 0.280 0.914 0.344

HETATM 4 C C2 1 -0.713 0.507 1.445

HETATM 5 C C2 1 0.294 2.415 0.017

HETATM 6 C C2 1 -3.528 -1.377 -0.274

HETATM 7 H C2 1 -1.019 -2.017 -0.518

HETATM 8 H C2 1 -0.534 0.523 -1.706

HETATM 9 H C2 1 1.012 2.576 -0.791

HETATM 10 H C2 1 0.646 2.974 0.894

HETATM 11 H C2 1 -0.265 0.540 2.440

HETATM 12 H C2 1 -1.625 1.119 1.434

HETATM 13 H C2 1 -3.638 -1.352 0.815

HETATM 14 H C2 1 -4.436 -0.990 -0.740

HETATM 15 H C2 1 -3.368 -2.415 -0.596

HETATM 16 H C2 1 -1.531 3.113 0.308

HETATM 17 O C2 1 -1.052 -0.865 1.155

HETATM 18 O C2 1 -2.453 -0.541 -0.707

HETATM 19 O C2 1 1.063 -0.692 -1.226

HETATM 20 O C2 1 1.597 0.540 0.686

HETATM 21 O C2 1 -0.956 2.939 -0.447

HETATM 22 O C2 1 3.419 -0.504 -0.609

HETATM 23 O C2 1 2.073 -1.872 0.714

HETATM 24 B C2 1 2.062 -0.652 -0.103

HETATM 25 H C2 1 1.228 -1.938 1.177

HETATM 26 H C2 1 3.446 0.241 -1.220

#REMARK Compound 3 – Implicit Solvation Geometry

#REMARK Sum of electronic and thermal Free Energies= -1246.453298

HETATM 1 C C3 1 -2.792 -1.420 -0.122

HETATM 2 C C3 1 -2.108 -0.183 -0.741

HETATM 3 C C3 1 -2.259 0.926 0.348

HETATM 4 C C3 1 -3.173 0.273 1.396

HETATM 5 C C3 1 -2.758 2.283 -0.176

HETATM 6 C C3 1 -4.749 -2.768 -0.331

HETATM 7 H C3 1 -2.185 -2.331 -0.194

HETATM 8 H C3 1 -2.605 0.071 -1.684

HETATM 9 H C3 1 -2.041 2.642 -0.919

HETATM 10 H C3 1 -2.777 3.005 0.650

HETATM 11 H C3 1 -2.904 0.563 2.414

HETATM 12 H C3 1 -4.233 0.501 1.220

HETATM 13 H C3 1 -5.034 -2.684 0.723

HETATM 14 H C3 1 -5.647 -2.837 -0.946

HETATM 15 H C3 1 -4.143 -3.674 -0.469

HETATM 16 H C3 1 -4.733 2.229 -0.176

HETATM 17 O C3 1 -2.952 -1.143 1.264

HETATM 18 O C3 1 -4.036 -1.610 -0.770

HETATM 19 O C3 1 -0.727 -0.356 -0.938

HETATM 20 O C3 1 -0.955 1.109 0.862

HETATM 21 O C3 1 -4.029 2.234 -0.837

HETATM 22 C C3 1 2.944 -1.429 0.033

HETATM 23 C C3 1 2.225 -0.321 0.833

HETATM 24 C C3 1 2.386 0.954 -0.053

HETATM 25 C C3 1 3.303 0.486 -1.197

HETATM 26 C C3 1 2.903 2.178 0.704

HETATM 27 C C3 1 4.950 -2.720 0.039

HETATM 28 H C3 1 2.367 -2.358 -0.033

HETATM 29 H C3 1 2.698 -0.223 1.819

HETATM 30 H C3 1 3.953 2.061 0.986

HETATM 31 H C3 1 2.309 2.309 1.620

HETATM 32 H C3 1 3.035 0.927 -2.158

HETATM 33 H C3 1 4.358 0.693 -0.980

HETATM 34 H C3 1 5.230 -2.463 -0.987

HETATM 35 H C3 1 5.851 -2.851 0.641

HETATM 36 H C3 1 4.377 -3.658 0.033

HETATM 37 H C3 1 1.930 3.327 -0.504

HETATM 38 O C3 1 3.090 -0.937 -1.291

HETATM 39 O C3 1 4.195 -1.671 0.650

HETATM 40 O C3 1 0.846 -0.540 0.964

HETATM 41 O C3 1 1.083 1.235 -0.531

HETATM 42 O C3 1 2.817 3.354 -0.109

HETATM 43 B C3 1 0.058 0.361 0.088

#REMARK Compound 4 – Implicit Solvation Geometry

#REMARK Sum of electronic and thermal Free Energies= -1246.450092

HETATM 1 C C4 1 3.185 1.046 0.016

HETATM 2 C C4 1 2.338 0.049 -0.804

HETATM 3 C C4 1 2.057 -1.124 0.188

HETATM 4 C C4 1 2.927 -0.777 1.405

HETATM 5 C C4 1 2.303 -2.528 -0.390

HETATM 6 C C4 1 5.406 1.897 0.195

HETATM 7 H C4 1 2.823 2.079 -0.050

HETATM 8 H C4 1 2.904 -0.263 -1.687

HETATM 9 H C4 1 1.642 -2.659 -1.252

HETATM 10 H C4 1 2.031 -3.282 0.359

HETATM 11 H C4 1 2.446 -1.052 2.347

HETATM 12 H C4 1 3.919 -1.245 1.354

HETATM 13 H C4 1 5.503 1.686 1.266

HETATM 14 H C4 1 6.379 1.786 -0.286

HETATM 15 H C4 1 5.050 2.927 0.062

HETATM 16 H C4 1 4.215 -2.952 -0.126

HETATM 17 O C4 1 3.065 0.656 1.379

HETATM 18 O C4 1 4.523 0.970 -0.439

HETATM 19 O C4 1 1.083 0.562 -1.172

HETATM 20 O C4 1 0.684 -1.014 0.501

HETATM 21 O C4 1 3.634 -2.749 -0.871

HETATM 22 C C4 1 -3.185 -1.046 0.016

HETATM 23 C C4 1 -2.338 -0.049 -0.804

HETATM 24 C C4 1 -2.057 1.124 0.188

HETATM 25 C C4 1 -2.927 0.777 1.405

HETATM 26 C C4 1 -2.303 2.528 -0.390

HETATM 27 C C4 1 -5.406 -1.897 0.195

HETATM 28 H C4 1 -2.823 -2.079 -0.050

HETATM 29 H C4 1 -2.904 0.263 -1.687

HETATM 30 H C4 1 -1.642 2.659 -1.252

HETATM 31 H C4 1 -2.031 3.282 0.359

HETATM 32 H C4 1 -2.446 1.052 2.347

HETATM 33 H C4 1 -3.919 1.245 1.354

HETATM 34 H C4 1 -5.503 -1.686 1.266

HETATM 35 H C4 1 -6.379 -1.786 -0.286

HETATM 36 H C4 1 -5.050 -2.928 0.062

HETATM 37 H C4 1 -4.215 2.952 -0.126

HETATM 38 O C4 1 -3.065 -0.656 1.379

HETATM 39 O C4 1 -4.523 -0.970 -0.439

HETATM 40 O C4 1 -1.083 -0.562 -1.172

HETATM 41 O C4 1 -0.684 1.014 0.501

HETATM 42 O C4 1 -3.635 2.749 -0.871

HETATM 43 B C4 1 -0.000 0.000 -0.334

#REMARK Compound 1 – Gas-Phase Geometry

#REMARK E0 Pucker State

#REMARK Sum of electronic and thermal Free Energies= -611.834095

HETATM 1 C C1 1 1.380 0.552 -0.063

HETATM 2 C C1 1 0.067 0.953 -0.739

HETATM 3 C C1 1 -0.990 0.090 0.024

HETATM 4 C C1 1 -0.253 -0.271 1.344

HETATM 5 C C1 1 -1.416 -1.146 -0.774

HETATM 6 C C1 1 3.055 -1.133 -0.120

HETATM 7 H C1 1 2.158 1.326 -0.112

HETATM 8 H C1 1 0.072 0.772 -1.815

HETATM 9 H C1 1 -0.558 -1.796 -0.960

HETATM 10 H C1 1 -1.826 -0.817 -1.740

HETATM 11 H C1 1 -0.790 0.097 2.218

HETATM 12 H C1 1 -0.097 -1.350 1.438

HETATM 13 H C1 1 2.955 -1.380 0.941

HETATM 14 H C1 1 3.301 -2.032 -0.687

HETATM 15 H C1 1 3.857 -0.392 -0.246

HETATM 16 H C1 1 0.114 2.606 0.320

HETATM 17 H C1 1 -2.021 1.747 -0.014

HETATM 18 H C1 1 -3.109 -1.326 0.154

HETATM 19 O C1 1 1.028 0.408 1.299

HETATM 20 O C1 1 1.826 -0.641 -0.656

HETATM 21 O C1 1 -0.191 2.347 -0.563

HETATM 22 O C1 1 -2.181 0.836 0.285

HETATM 23 O C1 1 -2.371 -1.916 -0.055

#REMARK Compound 1 – Gas-Phase Geometry

#REMARK 4T3 Pucker State

#REMARK Sum of electronic and thermal Free Energies= -611.833552

HETATM 1 C C1 1 1.411 0.298 0.271

HETATM 2 C C1 1 0.149 0.907 -0.391

HETATM 3 C C1 1 -1.007 -0.033 0.064

HETATM 4 C C1 1 -0.237 -1.302 0.466

HETATM 5 C C1 1 -2.077 -0.217 -1.016

HETATM 6 C C1 1 3.550 -0.565 -0.306

HETATM 7 H C1 1 1.903 0.991 0.965

HETATM 8 H C1 1 0.286 0.844 -1.476

HETATM 9 H C1 1 -1.680 -0.788 -1.864

HETATM 10 H C1 1 -2.395 0.765 -1.376

HETATM 11 H C1 1 -0.753 -1.890 1.229

HETATM 12 H C1 1 -0.033 -1.934 -0.412

HETATM 13 H C1 1 3.431 -1.480 0.285

HETATM 14 H C1 1 4.145 -0.776 -1.196

HETATM 15 H C1 1 4.065 0.193 0.302

HETATM 16 H C1 1 -0.661 2.263 0.711

HETATM 17 H C1 1 -2.524 0.265 1.255

HETATM 18 H C1 1 -3.142 -1.789 -0.447

HETATM 19 O C1 1 0.982 -0.823 1.035

HETATM 20 O C1 1 2.292 -0.075 -0.764

HETATM 21 O C1 1 -0.087 2.259 -0.074

HETATM 22 O C1 1 -1.604 0.572 1.215

HETATM 23 O C1 1 -3.256 -0.831 -0.476

#REMARK Compound 2 – Gas-Phase Geometry

#REMARK Sum of electronic and thermal Free Energies= -787.366417

HETATM 1 C C2 1 -1.224 -1.111 -0.233

HETATM 2 C C2 1 0.013 -0.346 -0.689

HETATM 3 C C2 1 0.226 0.727 0.440

HETATM 4 C C2 1 -0.663 0.205 1.600

HETATM 5 C C2 1 -0.151 2.155 0.011

HETATM 6 C C2 1 -3.612 -0.984 -0.408

HETATM 7 H C2 1 -1.233 -2.171 -0.515

HETATM 8 H C2 1 -0.163 0.111 -1.678

HETATM 9 H C2 1 0.458 2.415 -0.864

HETATM 10 H C2 1 0.101 2.851 0.818

HETATM 11 H C2 1 -0.049 -0.008 2.477

HETATM 12 H C2 1 -1.465 0.907 1.859

HETATM 13 H C2 1 -3.752 -0.878 0.673

HETATM 14 H C2 1 -4.389 -0.424 -0.936

HETATM 15 H C2 1 -3.695 -2.050 -0.672

HETATM 16 H C2 1 -1.898 1.536 -0.676

HETATM 17 O C2 1 -1.261 -1.047 1.170

HETATM 18 O C2 1 -2.364 -0.456 -0.825

HETATM 19 O C2 1 1.153 -1.138 -0.668

HETATM 20 O C2 1 1.587 0.680 0.748

HETATM 21 O C2 1 -1.549 2.344 -0.261

HETATM 22 O C2 1 3.013 0.438 -1.181

HETATM 23 O C2 1 3.244 -1.164 0.561

HETATM 24 B C2 1 2.308 -0.296 -0.138

HETATM 25 H C2 1 2.733 -1.794 1.080

HETATM 26 H C2 1 3.694 -0.142 -1.537

#REMARK Compound 3 – Gas-Phase Geometry

#REMARK Sum of electronic and thermal Free Energies= -1246.368296

HETATM 1 C C3 1 -2.995 -1.324 -0.470

HETATM 2 C C3 1 -2.056 -0.157 -0.764

HETATM 3 C C3 1 -2.145 0.729 0.520

HETATM 4 C C3 1 -2.687 -0.260 1.589

HETATM 5 C C3 1 -3.027 1.978 0.369

HETATM 6 C C3 1 -5.309 -1.905 -0.701

HETATM 7 H C3 1 -2.693 -2.274 -0.928

HETATM 8 H C3 1 -2.381 0.369 -1.676

HETATM 9 H C3 1 -2.639 2.568 -0.473

HETATM 10 H C3 1 -2.936 2.588 1.274

HETATM 11 H C3 1 -1.908 -0.474 2.324

HETATM 12 H C3 1 -3.589 0.116 2.086

HETATM 13 H C3 1 -5.449 -2.039 0.377

HETATM 14 H C3 1 -6.232 -1.526 -1.148

HETATM 15 H C3 1 -5.063 -2.879 -1.152

HETATM 16 H C3 1 -4.519 0.916 -0.362

HETATM 17 O C3 1 -3.015 -1.506 0.920

HETATM 18 O C3 1 -4.293 -0.952 -0.977

HETATM 19 O C3 1 -0.724 -0.559 -0.841

HETATM 20 O C3 1 -0.830 1.138 0.764

HETATM 21 O C3 1 -4.421 1.703 0.202

HETATM 22 C C3 1 3.149 -1.353 0.141

HETATM 23 C C3 1 2.324 -0.230 0.800

HETATM 24 C C3 1 2.412 0.952 -0.216

HETATM 25 C C3 1 3.385 0.439 -1.293

HETATM 26 C C3 1 2.819 2.291 0.411

HETATM 27 C C3 1 5.242 -2.467 0.330

HETATM 28 H C3 1 2.635 -2.321 0.154

HETATM 29 H C3 1 2.768 0.009 1.777

HETATM 30 H C3 1 3.884 2.309 0.672

HETATM 31 H C3 1 2.229 2.443 1.327

HETATM 32 H C3 1 3.068 0.723 -2.298

HETATM 33 H C3 1 4.411 0.790 -1.114

HETATM 34 H C3 1 5.521 -2.277 -0.713

HETATM 35 H C3 1 6.142 -2.472 0.953

HETATM 36 H C3 1 4.760 -3.456 0.390

HETATM 37 H C3 1 1.716 3.150 -0.887

HETATM 38 O C3 1 3.340 -0.998 -1.212

HETATM 39 O C3 1 4.393 -1.453 0.836

HETATM 40 O C3 1 0.972 -0.546 0.923

HETATM 41 O C3 1 1.110 1.090 -0.735

HETATM 42 O C3 1 2.587 3.360 -0.506

HETATM 43 B C3 1 0.132 0.274 0.027

#REMARK Compound 4 – Gas-Phase Geometry

#REMARK Sum of electronic and thermal Free Energies= -1246.365038

HETATM 1 C C4 1 3.388 1.038 -0.518

HETATM 2 C C4 1 2.254 0.064 -0.826

HETATM 3 C C4 1 2.153 -0.814 0.463

HETATM 4 C C4 1 2.854 0.061 1.538

HETATM 5 C C4 1 2.784 -2.208 0.331

HETATM 6 C C4 1 5.774 1.176 -0.705

HETATM 7 H C4 1 3.276 2.024 -0.985

HETATM 8 H C4 1 2.491 -0.522 -1.729

HETATM 9 H C4 1 2.304 -2.720 -0.514

HETATM 10 H C4 1 2.566 -2.784 1.238

HETATM 11 H C4 1 2.119 0.418 2.262

HETATM 12 H C4 1 3.664 -0.473 2.047

HETATM 13 H C4 1 5.916 1.289 0.375

HETATM 14 H C4 1 6.619 0.630 -1.134

HETATM 15 H C4 1 5.722 2.176 -1.163

HETATM 16 H C4 1 4.462 -1.453 -0.380

HETATM 17 O C4 1 3.416 1.223 0.872

HETATM 18 O C4 1 4.604 0.428 -0.999

HETATM 19 O C4 1 1.023 0.707 -0.927

HETATM 20 O C4 1 0.780 -0.967 0.689

HETATM 21 O C4 1 4.208 -2.203 0.187

HETATM 22 C C4 1 -3.388 -1.038 -0.518

HETATM 23 C C4 1 -2.254 -0.064 -0.826

HETATM 24 C C4 1 -2.153 0.814 0.463

HETATM 25 C C4 1 -2.854 -0.061 1.538

HETATM 26 C C4 1 -2.784 2.208 0.331

HETATM 27 C C4 1 -5.774 -1.176 -0.705

HETATM 28 H C4 1 -3.276 -2.024 -0.984

HETATM 29 H C4 1 -2.491 0.522 -1.729

HETATM 30 H C4 1 -2.305 2.720 -0.514

HETATM 31 H C4 1 -2.566 2.784 1.237

HETATM 32 H C4 1 -2.119 -0.418 2.262

HETATM 33 H C4 1 -3.663 0.474 2.048

HETATM 34 H C4 1 -5.916 -1.289 0.375

HETATM 35 H C4 1 -6.619 -0.630 -1.134

HETATM 36 H C4 1 -5.722 -2.176 -1.163

HETATM 37 H C4 1 -4.462 1.453 -0.380

HETATM 38 O C4 1 -3.416 -1.223 0.872

HETATM 39 O C4 1 -4.604 -0.428 -0.999

HETATM 40 O C4 1 -1.023 -0.707 -0.926

HETATM 41 O C4 1 -0.780 0.967 0.688

HETATM 42 O C4 1 -4.209 2.202 0.187

HETATM 43 B C4 1 0.000 0.000 -0.116


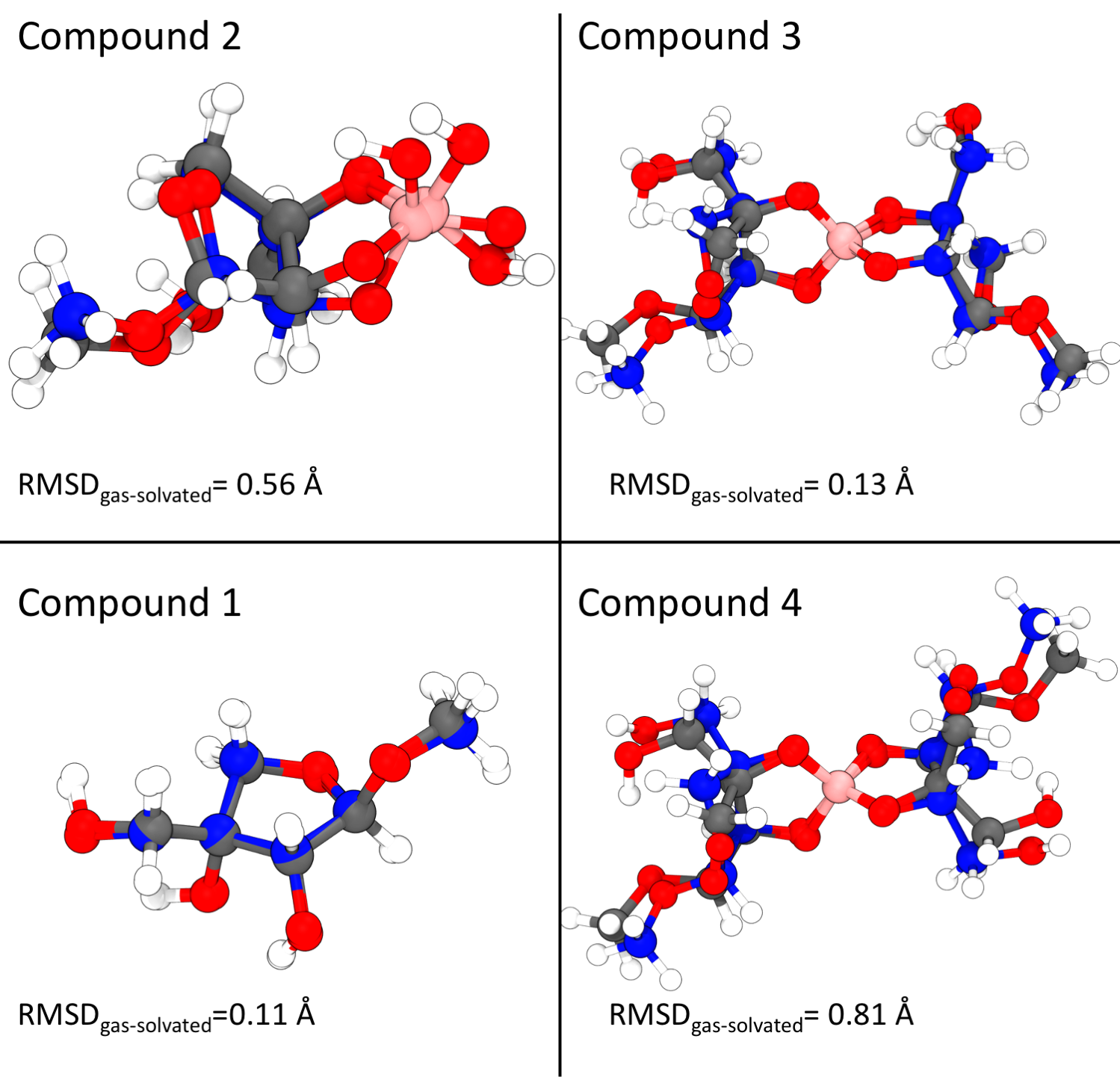


Figure S5: Comparison of implicit solvation and gas-phase optimized geometries. For each compound, the structures with carbons colored blue refer to the implicit solvation optimized geometries while the structure with carbons colored gray refer to the gas-phase optimized geometries.

**Supplementary References**

1. Ishii, T.; Yanagisawa, M., Synthesis, separation and NMR spectral analysis of methyl apiofuranosides. *Carbohydrate Research* **1998**, 4.

2. Ishii, T.; Ono, H., NMR spectroscopic analysis of the borate diol esters of methyl apiofuranosides. *Carbohydrate Research* **1999**, 4.

3. Fulmer, G. R.; Miller, A. J. M.; Sherden, N. H.; Gottlieb, H. E.; Nudelman, A.; Stoltz, B. M.; Bercaw, J. E.; Goldberg, K. I., NMR Chemical Shifts of Trace Impurities: Common Laboratory Solvents, Organics, and Gases in Deuterated Solvents Relevant to the Organometallic Chemist. *Organometallics* **2010,** *29* (9), 2176-2179.

4. Pierens, G. K., 1H and 13C NMR scaling factors for the calculation of chemical shifts in commonly used solvents using density functional theory. *J Comput Chem* **2014,** *35* (18), 1388-94.

5. Taubert, S.; Konschin, H.; Sundholm, D., Computational studies of 13C NMR chemical shifts of saccharides. *Phys Chem Chem Phys* **2005,** *7* (13), 2561-9.
